# Status of hormones and painkillers in wastewater effluents across several European states—considerations for the EU watch list concerning estradiols and diclofenac

**DOI:** 10.1007/s11356-016-6503-x

**Published:** 2016-03-29

**Authors:** P. Schröder, B. Helmreich, B. Škrbić, M. Carballa, M. Papa, C. Pastore, Z. Emre, A. Oehmen, A. Langenhoff, M. Molinos, J. Dvarioniene, C. Huber, K. P. Tsagarakis, E. Martinez-Lopez, S. Meric Pagano, C. Vogelsang, G. Mascolo

**Affiliations:** Research Unit Microbe–Plant Interactions (EGEN), German Research Center for Health and Environment GmbH, Helmholtz Zentrum Muenchen, Ingolstädter Landstr. 1, 85764 Neuherberg, Germany; Chair of Urban Water Systems Engineering, Technische Universität München, Munich, Germany; Faculty of Technology, University of Novi Sad, Novi Sad, Serbia; Department of Chemical Engineering, School of Engineering, University of Santiago de Compostela, Santiago de Compostela, Spain; Department of Civil Environmental Architectural Engineering & Mathematics, University of Brescia, Brescia, Italy; CNR—Istituto di Ricerca Sulle Acque, Bari, Italy; Turkish Atomic Energy Authority, Ankara, Turkey; Departamento de Química, Faculdade de Ciências e Tecnologia (FCT), Universidade Nova de Lisboa (UNL), Caparica, Portugal; Sub-department of Environmental Technology, Wageningen University of Agrotechnology & Food Sciences, Wageningen, The Netherlands; University of Valencia, Valencia, Spain; Kaunas University of Technology, Kaunas, Lithuania; Business and Environmental Economics Technology Lab (BETECO), Department of Environmental Engineering, Democritus University of Thrace, Xanthi, Greece; University of Murcia, Murcia, Spain; Namık Kemal Üniversitesi, Tekirdağ, Turkey; Norwegian Institute for Water Research (NIVA), Oslo, Norway

**Keywords:** Diclofenac, Ethinylestradiol, Emerging pollutants, Effluent quality, EU watch list, Pollutant removal, Advanced technologies

## Abstract

Present technologies for wastewater treatment do not sufficiently address the increasing pollution situation of receiving water bodies, especially with the growing use of personal care products and pharmaceuticals (PPCP) in the private household and health sector. The relevance of addressing this problem of organic pollutants was taken into account by the Directive 2013/39/EU that introduced (i) the quality evaluation of aquatic compartments, (ii) the *polluter pays* principle, (iii) the need for innovative and affordable wastewater treatment technologies, and (iv) the identification of pollution causes including a list of principal compounds to be monitored. In addition, a watch list of 10 other substances was recently defined by Decision 2015/495 on March 20, 2015. This list contains, among several recalcitrant chemicals, the painkiller diclofenac and the hormones 17β-estradiol and 17α-ethinylestradiol. Although some modern approaches for their removal exist, such as advanced oxidation processes (AOPs), retrofitting most wastewater treatment plants with AOPs will not be acceptable as consistent investment at reasonable operational cost. Additionally, by-product and transformation product formation has to be considered. The same is true for membrane-based technologies (nanofiltration, reversed osmosis) despite of the incredible progress that has been made during recent years, because these systems lead to higher operation costs (mainly due to higher energy consumption) so that the majority of communities will not easily accept them. Advanced technologies in wastewater treatment like membrane bioreactors (MBR) that integrate biological degradation of organic matter with membrane filtration have proven a more complete elimination of emerging pollutants in a rather cost- and labor-intensive technology. Still, most of the presently applied methods are incapable of removing critical compounds completely. In this opinion paper, the state of the art of European WWTPs is reflected, and capacities of single methods are described. Furthermore, the need for analytical standards, risk assessment, and economic planning is stressed. The survey results in the conclusion that combinations of different conventional and advanced technologies including biological and plant-based strategies seem to be most promising to solve the burning problem of polluting our environment with hazardous emerging xenobiotics.

**Electronic supplementary material:**

The online version of this article (doi:10.1007/s11356-016-6503-x) contains supplementary material, which is available to authorized users.

## Introduction and demand

Across Europe, most people do not know where their drinking water comes from, and they are not aware of how big the efforts are to allow the performance of the most normal daily action, namely to open the tap and to consume clean, clear, and pure water. Still, it is the extremely high quality of our drinking water that guarantees the healthy life we lead. In fact, to provide unpolluted water as a resource for drinking water supply, food production but also other aspects of daily life will remain one of the major challenges for Europe in the near future. Novel emergent organic compounds (pharmaceuticals, industrial chemicals, personal care products, and others) pose a threat to our water reserves (Heberer 2002a, b; Kasprzyk-Hordern et al. 2008). These anthropogenic substances, often addressed as micropollutants that may adversely affect drinking water quality, are most typically polar to semipolar organic compounds detected at concentrations in the picogram per liter to microgram per liter range (Benner et al. 2013). Contamination of drinking water resources (surface water and groundwater) with these micropollutants raises important questions related to human health, ecology, and economic impacts (Benner et al. 2013). Among sources that are considered responsible for the occurrence of micropollutants in surface and groundwater, effluents of municipal wastewater treatment plants (WWTPs) are frequently pinpointed as the most important (Ternes 1998; Zuccato et al. 2006; Kasprzyk-Hordern et al. 2008). Whereas well-assessed treatment strategies exist for classical issues in WWTPs such as removal of biodegradable organic substances, nutrients (phosphorus and nitrogen), detergents, and even microorganisms, polar and semipolar micropollutants are not or only incompletely removed by these technologies. Hence, with the growing number of micropollutants being identified in surface water and groundwater, novel remediation and management strategies are needed to provide cost-effective and sustainable treatment solutions across Europe.

Since the majority of all significant water bodies, lakes, and streams are shared between several European countries, the European Union (EU) has to find a common strategy for remediation of micropollutants and to set limits for effluents from WWTPs. Furthermore, it will be necessary to expand the scope of water protection to all waters, surface waters and groundwater, to achieve satisfactory status for all waters by a set deadline, and to delegate water management to regional authorities based on river catchments. This is in part proposed by the European Water Framework Directive (see below: international conventions and agreements), which has already been implemented in most EU countries. The relevance of addressing the problem of organic pollutants was also taken into account by the Directive 2013/39/EU that introduced (i) the quality evaluation of aquatic compartments, (ii) the *polluter pays* principle, (iii) the need for innovative and affordable wastewater treatment technologies, and (iv) the identification of pollution causes including a list of principal compounds to be monitored.

**Fig. 1:**
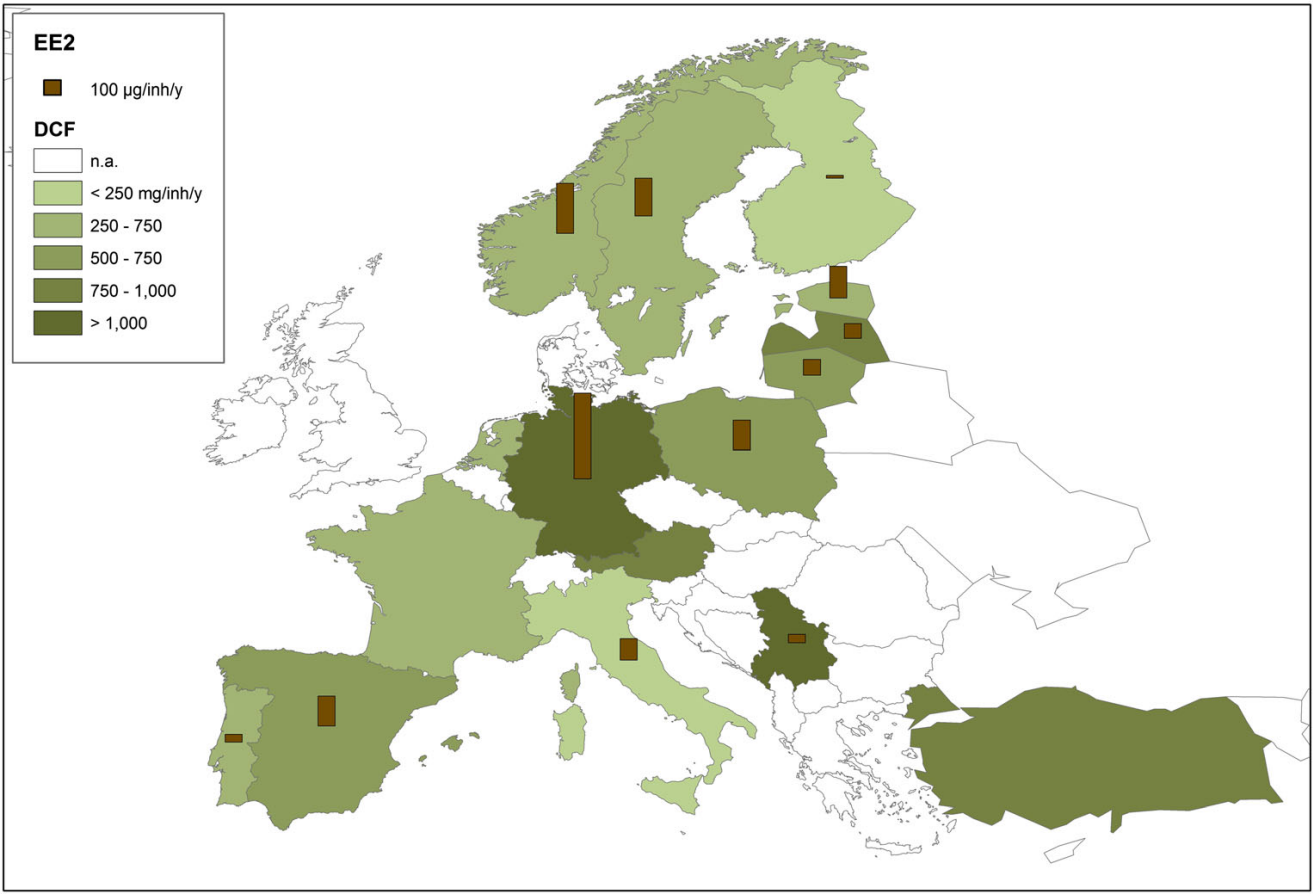
Graphical representation of DCF and EE2 consumption levels across the EU

Among thousands of micropollutants, not everything that can be measured is worth measuring, and not everything worth measuring is measurable. With regard to pharmaceutically active compounds, those to be monitored in natural waters should be related to prescription and nonprescription practices in each country (compare Fig. 1). It is very important to develop a ranking system to prioritize pharmaceutically active compounds considering the following four criteria: (a) occurrence (prevalence, frequency of detection), (b) highest percentages of excretion, (c) removal in treatment plants, and (d) ecological effects (bioaccumulation, ecotoxicity).

From the large number of micropollutants that fit this scheme, diclofenac (2-(2-(2,6-dichlorophenylamino)phenyl)acetic acid, DCF) and the estrogenic hormones 17β-estradiol (1,3,5(10)-estratrien-3,17β-diol; E2) and 17α-ethinylestradiol (19-nor-17α-pregna-1,3,5(10)-trien-20-in-3,17-diol; EE2) have recently been included in the updated watch list of 10 other substances defined by Decision 2015/495 on March 20, 2015.

Diclofenac is a widely used nonsteroidal anti-inflammatory drug used as a painkiller prescribed as pills or ointments and among the most frequently detected pharmaceuticals in WWTP effluents, in microgram per liter concentrations (Verlicchi et al. 2012). E2 is a primary female sex hormone and key regulator of the estrous and menstrual female reproductive cycles, whereas EE2 is a synthetic, bioactive estrogen used in many formulations of combined oral contraceptive pills. Both estrogens are detected in WWTPs in the lower nanogram per liter concentrations and are known to cause endocrine-disrupting effects in the biota (Forrez et al. 2009). Again, WWTP effluents are considered the main source of estrogens in the environment (Snyder et al. 2001).

Using these three selected micropollutants from the EU watch list as representatives, the aim of this review is to summarize current problems and solutions in several EU countries and critically evaluate the viability of various treatment methods for the removal of micropollutants from wastewater.

The North Sea Conference on Co-operation in dealing with pollution of the North Sea by oil and other harmful substances (Bonn, 1983), stimulated public awareness to the topic of water quality for the first time. Still, it took almost a decade until the Hague Declaration on the future European Community groundwater policy was ratified at the EC Ministerial Meeting on 26.-27.11.91. Another decade went by until the Agenda 21 requested that quantitative and qualitative discharge standards for municipal and industrial effluents should be established and applied by the year 2000. This recommendation included the proposal to revise Directive 76/464/EEC (Dangerous Substances in Water) and the Directive No. 96/61 EC on Integrated Pollution Prevention and Control (IPCC 1996), as well as Directive 93/793/EEC on environmental risk from chemicals (testing the ecotoxicity of listed priority chemicals). Nowadays, updated European framework legislation promotes the reduction of micropollutants. The ETAP (Environmental Technologies Action Plan) of the European Union claims urgent action for better water quality and protection of our natural resources. High priority is also given to environmentally sound water treatment technologies that will reduce greenhouse gases, recycle materials and provide all partner countries with affordable technologies. The discussion paper on water issues is very specific about novel green technologies to be adopted in this respect (http://europa.eu.int/comm/environment/etap/pdfs/etapwaterissuefr.pdf). Substantial political concern exists that water pollutants have to be monitored and removed. However, our knowledge of xenobiotics control or degradation has hardly gone beyond scratching the surface and confirming the importance of the problem. Finally, the EU enhanced the list of dangerous compounds and put estradiols and diclofenac on the Watchlist (Directive 2013/39/EU). The substances (diclofenac and two hormones: 17β-estradiol (E2) and 17α-ethinylestradiol (EE2)) shall be monitored by the EU member states in their surface waters for a maximum of four years. In addition, environmental quality standard values of 100 ng L^−1^ for inland waters and 10 ng L^−1^ for coastal water were proposed for diclofenac. Although such political decisions are very helpful to increase the public awareness of water pollution problems, our general behavior towards water protection and water pricing is ambiguous.

## National inventories

The availability of data on the daily use of pharmaceuticals in the EU is scattered and incomplete for recent years. A comprehensive view may be possible for the years between 2005 and 2011 where data from several countries can be compared (Fig. 1). Diclofenac and EE2 consumption rates vary greatly between and also within countries. According to literature data in different countries around the world (Ternes 1998; Grung et al. 2007, 2008; Carballa et al. 2005, 2008; Ferrari et al. 2011; INFARMED 2012), the annual consumption of DCF varies between 195 and 940 mg per inhabitant; as for EE2, it varies between 20 and 580 μg per inhabitant, respectively. However, in Serbia with a population of 7.2 million in 2012, the annual consumption of DCF and EE2 was equal to 8650 and 0.39 kg per year, respectively, while the consumption of DCF and EE2 per inhabitant was estimated to be 1197 mg and 50 μg per year per inhabitant, respectively (Radonjić and Šipetić 2012). In Germany, for DCF and EE2, respectively, consumption quantities of active ingredients in human medicine were estimated to be 1033 mg and 600 μg per inhabitant per year (SRU 2007), while the DCF consumption rate in Turkey for the years 2009 and 2013 was 950 and 985 mg per inhabitant per year, respectively (Sari et al. 2014), and 440 mg per inhabitant and year in The Netherlands (Oosterhuis et al. 2013).

For the calculation of drug consumption in several EU countries for 2011 (Supplemental Table 1), the concept of “defined daily dose” (DDD) was used, i.e., the assumed average maintenance dose per day for a drug used for its main indication in adults expressed as DDD/1000 inhabitants/day, as proposed by the WHO Collaborating Centre for Drug Statistics Methodology (http://www.whocc.no/). Comparing the consumption data, obviously, the DCF and EE2 are among the most popular and most consumed medicine products.

Recent analysis of the consumption of DCF and EE2 in three Baltic States for the 2009–2013 period revealed that the demand for drugs that affect the musculoskeletal system has increased by 4.9 % and the demand for drugs that affect the urogenital system and sex hormones has increased by 9.85 %. The sales of diclofenac were equal to 30.3 % of all the sales in accordance with the Anatomical Therapeutic Chemical (ATC) subgroup; the sales of EE2 in its subgroup were equal to 11.6 % (Estonian State Agency of Medicines 2013; Baranauskaite and Dvarioniene 2014).

## Occurrence of analgesics and hormones in WWTP’s effluents and surface waters

Various studies over recent years have shown that treated municipal wastewater contributes significantly to water pollution by micropollutants (Hollender et al. 2009; Jelic et al. 2012; Kasprzyk-Hordern et al. 2009; Ternes 1998; Verlicchi et al. 2012). This is a consequence of the increasing number of prescribed medicaments and of the fact that state-of-the-art sewage treatment plants are obviously not designed to remove personal care products and pharmaceuticals (PPCPs) from the wastewater they receive from households and hospitals. DCF, E2, and EE2 have been detected in both WWTPs (influents (WWTP-I) and effluents (WWTP-E)) and surface waters in the range of low microgram per liter to few nanogram per liter levels (Table 1).

**Table 1:** Concentrations of painkillers and hormones recorded in the aquatic environment during the last decade (2003–2013)

Compound	Type of water	Conc. (ng L^−1^)	Country	Citation
DCF	WWTP-E	≤1612	Portugal	Salgado et al. (2010)
	WWTP-I	4534–38,674	Portugal	Salgado et al. (2012)
	WWTP-I	1020	Italy	Patrolecco et al. (2013)
	WWTP-E	507	Italy	Patrolecco et al. (2013)
	WWTP-E	5450	Italy	Andreozzi et al. (2003)
	WWTP-E	250–5450	France, Italy, and Greece	Jiskra (2008)
	WWTP-E	2200	Germany	Letzel et al. (2009)
	WWTP-E	310–930	Switzerland	Jiskra (2008)
	WWTP-E	290	UK	Thomas and Hilton (2004)
	WWTP-E	125	UK	Roberts and Thomas (2005)
	WWTP-E	99	Switzerland	Tixier et al. (2003)
	WWTP-E	91	UK	Ashton et al. (2004)
	WWTP-E	0.14	Finland	Bignert et al. (2013)
	SW	261	UK	Kasprzyk-Hordern et al. (2008)
	SW	140	Germany	Letzel et al. (2009)
	SW	94	China	Huang et al. (2011)
	SW	89	Germany	Heberer (2002b)
	SW	16–65	Finland	Bignert et al. (2013)
	SW	35	Finland	Vulliet et al. (2011)
	SW	10–16	Italy	Marchese et al. (2003)
	SW	1.6	Italy	Loos et al. (2013)
	SW	4–260	The Netherlands	RIWA (2014)
	SW	10–120	Belgium	RIWA (2014)
	GW/DW	6	Germany	Heberer (2002b)
E2	WWTP-I	≤97	Portugal	Salgado et al. (2012)
	WWTP-I	64	Belgium	Forrez et al. (2009)
	WWTP-E	15–27	Germany	Carballa et al. (2004)
	WWTP-E	17	USA	Wright-Walters and Volz (2007)
	WWTP-E	<10	Sweden	Bigner et al. (2013)
	SW	9.5	Italy	Pojana et al. (2007)
	SW	9	Italy	Viganò et al. (2008)
	SW	1	Italy (Rome)	Baronti et al. (2000)
	SW	<1	The Netherlands	RIWA (2014)
EE2	WWTP-I	≤39	Portugal	Salgado et al. (2012)
	WWTP-I	106	Belgium	Forrez et al. (2009)
	WWTP-E	<1	Spain	Carballa et al. (2004)
	WWTP-E	0.04	Sweden	Bigner et al. (2013)
	SW	11	Italy	Pojana et al. (2007)
	SW	0.04	Italy (Rome)	Baronti et al. (2000)
	SW	<500	The Netherlands	RIWA (2014)

*WWTP-I* wastewater treatment plant influent, *WWTP-E* wastewater treatment plant effluent, *SW* surface water, *DW* drinking water, *GW* groundwater

One of the first compilations on this topic was a German study detecting diclofenac among 55 pharmaceuticals and 9 of their metabolites in the discharge of 49 sewage treatment plants as well as in their effluents in concentrations of up to several micrograms per liter (Ternes 1998). In the UK, DCF was detected in estuaries at concentrations up to 125 ng L^−1^ (Thomas and Hilton 2004). Another study reported that 27 out of 32 pharmaceutical substances and 4 of 5 metabolites were detected in the effluents of European wastewater treatment plants and that surface water peak values exceeded 1 μg L^−1^ (Larsen et al. 2004). During an EU-wide monitoring survey on emerging polar organic contaminants in wastewater treatment plant effluents, DCF was found at an average concentration of 49.5 ng L^−1^, while the highest concentration found was 174 ng L^−1^ (Loos et al. 2013).

This pollution in the effluents leads to contamination of surface water as has been proven in several novel studies. Levels of target compounds were in the nanogram per liter range but concentrations of some of them exceed 1 μg L^−1^ (including DCF) with fairly high concentrations of 1.3 μg L^−1^ and even 20.1 μg L^−1^, respectively (Petrović et al. 2014), reflecting the consumption of PhACs by the residents of Novi Sad, the second largest town in Serbia. In recent Spanish investigations, DCF seasonal behavior was also monitored north of El Albujón, till the Mar Menor Lagoon into the Mediterranean Sea, where even concentrations of 50 ng L^−1^ were detected (Moreno-González et al. 2014). Analogously, along the Turia River, which flows into the Mediterranean Sea at some kilometers farther at north than El Albujón, a very consistent amount of DCF was determined (Carmona et al. 2014). In this case, 3500 ng L^−1^ was detected in the water, whereas a contamination of sediments of 100 ng g^−1^ was determined.

The Turia case represents an excellent example for the impact on human uses of water contaminated by DCF, considering that water obtained after osmotic treatment, then used as “drinkable” water, still had a concentration of 18 ng L^−1^ of this pharmaceutical. Concentrations of painkillers and hormones recorded in the aquatic environment during the last decade (2003–2013) are given in Table 1.

As for estrogens, concentrations of 1–500 ng L^−1^ have been recorded in untreated municipal wastewater, with the distribution of concentrations generally following the pattern E1 > E2 > E3 > EE2 (Racz and Goel 2009). Interestingly, concentrations of 1–500 ng L^−1^ have also been reviewed by the same authors in the effluents of wastewater treatment facilities, indicating that elimination of these endocrine substances is insufficient in many if not all treatment systems.

## Progress in detection and identification

The detection and identification of PPCPs in environmental samples can be divided into three categories, namely quantitative targeted analysis employing reference standards, suspects screening without reference standards, and nontargeted screening (Krauss et al. 2010; Kind and Fiehn 2010; Little et al. 2011). Quantitative target analysis is the most common approach, in which only a number of previously selected, and often regulated, compounds are determined and the method is only validated for such compounds. For the monitoring of the target compounds DCF, EE2, and E2 in water samples, preconcentration is required prior to analysis. Currently, solid-phase extraction (SPE) is the most widely used procedure to extract and concentrate pharmaceuticals and other organic pollutants from environmental samples. In the specific case of DCF, acidification of the aqueous sample is frequently used to facilitate more efficient recovery of the target molecule from natural samples (Table 2). When the adopted analytical technique is based on gas chromatography (GC) coupled with mass spectrometry (GC-ion trap-MS/MS, GC-MS, or GC-MS-SIM), derivatization is necessary (methylation, terbutylation, etc.) to enable separation and detection. These operations are not necessary when final analysis is performed with LC-MS/MS. In any case, pretreatment and derivatization will enhance the overall difficulty of the analysis, and its final net cost for the respective additional preparative works, without a significant difference in terms of limits of quantification (LOQ). Consequently, and specifically since coelution occurs, several labs have proposed to omit this preconcentration step and begun to search for other solutions.

**Table 2:** Relevant information related to preconcentration steps and analysis of environmental water samples for diclofenac, E2, and EE2 determination. Costs listed refer to the different analytical options, without considering those related to instrument investment or the possibility, for each method, to be capable of determining several compounds simultaneously (multiresidual analysis). In any case, limiting the determination only to a restricted number of target compounds could be considered a too simplistic approach which might not be useful to fully take advantage of the potentialities of the instrumentation nowadays available

Sample	Preparation steps	Recovery (%)	Analytical technique	LOQ (μg L^−1^)	Analysis time and difficulty^a^	Analysis costs (€/sample)^b^	Reference
Diclofenac							
Wastewater influent and effluent	1. Filtration 2. SPE preconcentration 3. Derivatization	100	GC-MS/MS	0.05	+++	40–60	Carballa et al. (2004, 2005, 2007)
raw industrial and municipal wastewater, surface, ground, drinking water	1. Acidification 2. SPE pre-concentration 3. IS addition	55–116	LC-ESI-MS/MS	0.012–0.02	++	30–50	Gros et al. (2006a, b, 2009, 2012), Petrović et al. (2014)
Wastewater influent and effluent, groundwater	1. Filtration 2. SPE preconcentration 3. Derivatization	55–100	GC-MS	0.025	+++	40–60	Ternes (1998, 2001), Ternes et al. (2003)
Wastewater influent and effluent, basin water	1. Acidification 2. IS addition 3. SPE preconcentration	100	LC/ESI-MS/MS	>0.03	++	30–50	Sacher et al. (2008), Oosterhuis et al. (2013)
Wastewater influent and effluent	1. Acidification 2. IS addition 3. SPE preconcentration	100	UHPLC-MS/MS	0.05–0.14	++	30–50	Gracia-Lor et al. (2010, 2011)
River water, WWTP effluent	1. Addition of deuterated standards 2. Acidification 3. SPE preconcentration 4. IS addition	99	LC/ESI/MS	0.02	++		Letzel et al. (2009)
Wastewater influent and effluent	1. Acidification 2. SPE preconcentration 3. Derivatization	65–85	GC/ion trap-MS/MS	0.12	+++	40–60	Serrano et al. (2011)
River, wastewater influent and effluent	1. Filtration 2. SPE preconcentration 3. Derivatization	56–112	UHPLC-MS/MS	0.006–0.012	+++	40–60	Huang et al. (2011)
E2 and EE2							
Surface water and wastewater	1. Filtration (1.5 μm) 2. SDB-XC disk extraction 3. SPE (C18 and NH2) 4. HPLC elution 5. Derivatization	88–92	GC/ion trap-MS/MS	0.1–2.4	+++	40–60	Belfroid et al. (1999)
Wastewater influent and effluent, rivers	1. Filtration (1.5 μm) 2. SPE preconcentration 3. Addition of IS	80–92	LC/ESI-MS/MS	0.008–0.8	++	30–50	Baronti et al. (2000)
Wastewater influent and effluent, anaerobic digester influent and effluent	1. Filtration (1.5 μm) 2. SPE purification/preconcentration 3. Derivatization	82–84	GC/ion trap-MS/MS	1	+++	40–60	Ternes (1998), Carballa et al. (2004, 2005, 2007)
Synthetic, wastewater influent and effluent, surface waters	1. Filtration (1.5 μm) 2. MeOH and IS addition 3. SPE purification/preconcentration 4. Derivatization	79–100	GC/ion trap-MS/MS	3–20	+++	40–60	Quintana et al. (2004)
Surface water, wastewater influent and effluent	1. Filtration	65–105	LC/LC-MS/MS	0.002–0.003	+++	20–40	Gorga et al. (2013)

^a^+: low, ++: moderate, +++: high

^b^Analysis cost was estimated including the cost of the column (lasting about 500 injections) and SPE cartridge and amortization of instrumentation (lasting 5 years)

Often there is lack of information on analyzed samples because only user-defined MS/MS transitions are saved in the method and compounds in the sample that are not specified beforehand remain unknown. Employment of the MS/MS techniques for quantitative target analysis has also some drawbacks and limitations, namely (i) methods are typically limited to about 100–150 target compounds depending on chromatographic separation under the constraints of having at least two transitions per compound; (ii) for some compounds, only nonspecific transitions might occur such as the neutral loss of H_2_O or CO_2_, which are also common for matrix interferences; and (iii) for some analytes, especially those of low molecular weight, only one transition is present.

When analyzing sewage sludge, an additional step is necessary for exhaustive determination of DCF, E2, and EE2. Namely, the first step in pretreatment usually applied involves extraction of the target compounds from a solid sample by pressurized liquid extraction (PLE, Radjenović et al. 2009), microwave assisted extraction (MAE, Cortazar et al. 2005; Rice and Mitra 2007), or ultrasound sonification (US, Gatidou et al. 2007). In addition, an extensive cleaning of the obtained extract to avoid any matrix interference will remove organic and inorganic coextractives, before they might interfere with analyte separation and detection causing background noise in GC-MS analysis and signal suppression and/or enhancement in LC-MS analysis.

After application of one of the mentioned extraction techniques (PLE, MAE, or US) as the first pretreatment step to solid matrices, the next steps involved are presented in Table 2. Which of the listed methodologies will be selected depends on the type of analyte and particular techniques available in the laboratory.

In Table 2, the main published procedures for analyzing DCF, E2, and EE2 in environmental water samples are compiled. It becomes clear that for both DCF and the estrogen determination by GC-MS necessarily involves an additional derivatization step (e.g., by methylation, *tert*-butyldimethylsilyl, with N-methyl-N-(trimethylsilyl)trifluoroacetamide, etc.) due to the polarity of the compounds. Determination by LC-MS is indeed simpler and can even be automated provided that an online SPE can be used to reach the low detection limits that are frequently required (Patrolecco et al. 2013).

Another issue worth considering is the presence in environmental aqueous samples, together with target pharmaceutical compounds, of other compounds that are practically linked to the selected targets, namely metabolites and transformation products (TP). The determination of such compounds is not straightforward due to the lack of relevant mass spectrometric data available in LC-MS/MS methods, namely the precursor ion mass, the product ion masses (quantifier ion and qualifier ion), and the collision energy voltage. Therefore, an approach that is not based on the selectivity of the MS/MS mode but that employs high-resolution MS (HRMS) allowing the detection in scan mode would be much more beneficial.

In nontarget screening analysis, unknown components in the sample chromatogram are extracted from tentatively identified compounds (TIC), using special deconvolution software that detects the ions filtering them out from the background. For this type of experiment, the employment of HRMS(/MS) is reported to be the only effective technique to be used (Krauss et al. 2010; Nurmi et al. 2012; Godfrey and Brenton 2012). Indeed, a structure proposition for a peak detected by HRMS and MS/MS spectra involves several work-intensive data and expert processing steps (Krauss et al. 2010; Nurmi et al. 2012; Kind and Fiehn 2010; Little et al. 2011, 2012; Amorisco et al. 2013).

It is evident that nontarget screening analysis is incapable of revealing all compounds in the sample, causing possible false negative results. This is due to the inherent nature of LC-MS analysis, since both, chromatography and ionization always exclude some of the compounds. As a very useful evaluation tool for possible candidates, HRMS is ideal when combined subsequently with a powerful structure elucidation technique like nuclear magnetic resonance spectroscopy (NMR, De Laurentiis et al. 2014). An efficient modern method for both target and nontarget screening analysis for DCF is the hyphenation of hydrophilic interaction chromatography (HILIC) with RPLC coupled with highly accurate MS, such as TOF-MS.

With the set of detection methods discussed here, the analyst has a powerful tool for comprehensive and simultaneous analysis of compounds in a wide range of polarity, including the estrogens, DCF, and their transformation products (Rajab et al. 2013).

## Conventional treatment systems and their shortcomings

Conventional WWTPs are designed to limit the discharges of organic carbon, nitrogen, phosphorus, and pathogens to the aquatic environment. To do so, WWTPs apply a primary, a secondary, and an optional tertiary treatment process. During primary treatment, coarse solids are separated from the liquid stream and micropollutants are removed mainly by chemical and mechanical separation. The sorption of micropollutants onto solids depends basically on their physicochemical properties, such as lipophilicity or acidity. Two types of coefficients have been mostly used to determine the sorption effectiveness: the octanol-water partition coefficient (*K*_ow_) and the organic carbon partition coefficient (*K*_oc_). Log *K*_ow_ < 2.5 indicates a low sorption potential, 2.5 < log *K*_ow_ < 4 indicates a medium sorption potential, while log *K*_ow_ > 4 indicates a high sorption potential (Rogers 1996). However, some limitations have been found in the literature (Holbrook et al. 2004; Lai et al. 2000) for the applicability of these coefficients to explain the sorption behavior of some micropollutants, because acidity determined by functional groups also plays a significant role in sorption behavior. Therefore, the solid-water distribution coefficient (*K*_d_), defined as the ratio between the concentrations of a substance in the solid and in the aqueous phase at equilibrium conditions, has been proposed as the most suitable parameter (Schwarzenbach et al. 2003; Ternes et al. 2004a, b; Joss et al. 2005). This coefficient takes into account the two main sorption mechanisms absorption (hydrophobic interactions characterized by the *K*_ow_ value, relevant for neutral compounds) and adsorption (electrostatic interactions related to the substance tendency to be ionized or dissociated in aqueous phase, characterized by the dissociation constant, p*K*_a_). At pH above the p*K*_a_, phenolic hydroxyl or carboxyl groups dissociate and become negatively charged (Schäfer et al. 2011). DCF, for example, with a p*K*_a_ > 4 is negatively charged in municipal WWTP effluents, while E2 and EE2 are still in their neutral form. Table 3 summarizes these properties for the compounds under consideration. It can be observed that the three substances show a medium tendency to sorb onto solids, and consequently, only intermediate removal (20–45 %) has been obtained during primary treatment (Carballa et al. 2005; Behera et al. 2011).

**Table 3:** Molecular properties of the compounds under consideration

Compound	Molecular weight (g mol^−1^)	Molecular width (Å)	Log *K*_ow_	p*K*_a_	Log *K*_d_	*k*_biol_ for CAS L/(g_ss_ day)
DCF	296.2	5.95^a^	4.5–4.8^a, b^	4.0–4.5^b^	1.2^c^–2.1^d^	≤0.1^e^
E2	272.4	5.21^a^	3.9–4.0^a, f^	10.4	2.5–3.5	300–800
EE2	296.4		2.8–4.2^f^	10.5–10.7	2.3–2.8^c^	7–9^g^

^a^Drewes et al. (2005)

^b^Yang et al. (2011)

^c^Ternes et al. (2004a, b)

^d^Radjenović et al. (2009)

^e^Joss et al. (2006)

^f^Schäfer et al. (2011)

^g^Suárez et al. (2008)

The most commonly applied secondary treatment in WWTPs is the conventional activated sludge process (CAS), where both organic matter and nutrients are biologically removed. In this step, removal of a parent compound occurs by different mechanisms: a) stripping by aeration; b) sorption to particles or biomass; and c) biotransformation/biodegradation. Stripping is not significant for DCF, EE2 or E2 due to their high molecular mass and therefore low volatility (Radjenović et al. 2009). As described in the previous paragraph, sorption to sewage sludge is moderate, and therefore, biological transformation is the most likely mechanism responsible for micropollutant elimination in WWTPs. Although the microbiota developed in WWTPs may have been exposed to a plethora of micropollutants for a long time, the effective biological removal of these substances is conditioned by singular factors. Some of these factors are micropollutant-related, such as chemical structure or functional groups. In general, linear compounds with short side chains, unsaturated aliphatic compounds, and compounds possessing electron donating functional groups are easily degradable (Luo et al. 2014). The biodegradability of organic compounds is commonly classified according to their kinetic reaction rate (*k*_biol_). Suarez et al. (2010) have defined four groups of substances according to their biodegradability based on grams of suspended solids (ss) and days:

- Very highly degradable: *k*_biol_ > 5 L/(g_ss_ day)
- Highly degradable: 1 < *k*_biol_ < 5 L/(g_ss_ day)
- Moderate degradable: 0.5 < *k*_biol_ < 1 L/(g_ss_ day)
- Hardly degradable: *k*_biol_ < 0.5

From the data compiled in Table 3, only E2 and EE2 can be identified as very highly degradable, while DCF is very recalcitrant. However, it should be considered that these degradation constants are usually determined in lab-scale experiments and the operational conditions in WWTPs might be different. In fact, there is evidence that some operating parameters, such as hydraulic retention time (HRT), solid retention time (SRT), redox conditions, and temperature may affect micropollutant removal. HRT is the time that allows for biodegradation and sorption (Luo et al. 2014). Micropollutants having slow/intermediate kinetics will experience less effective biotransformation at shorter HRT or increasing loading rates (Fernandez-Fontaina et al. 2012). However, for E2 and EE2, the effect of this parameter is minor. Extended SRT, facilitating the buildup of slowly growing microbes, such as nitrifying bacteria, will enhance the elimination of micropollutants (Clara et al. 2005; Suarez et al. 2010, 2012; Silva et al. 2012; Luo et al. 2014), but beyond 25–30 days, this parameter is not significant anymore. This influence is clear for E2 and EE2, but contradictory results have been published for DCF. According to the findings of Joss et al. (2005), the elimination rates of DCF did not improve even when extreme SRT (more than 60 days) was applied. In contrast, promoted removal rates for DCF with increasing SRT were reported by Nikolaou et al. (2007), Stasinakis et al. (2010), Falas et al. (2012), Fernandez-Fontaina et al. (2012), and Falas et al. (2013). However, extremely high SRT (>150 days) is unrealistic in conventional WWTPs with activated sludge process. Regarding redox conditions, different removal efficiencies have been observed for anaerobic, anoxic, and aerobic conditions (Joss et al. 2004). Overall, aerobic conditions are preferable for estrogen removal (Silva et al. 2012), while anoxic and anaerobic conditions might be slightly better for DCF (Zwiener and Frimmel 2003; Vieno and Sillanpää 2014). Finally, higher temperatures positively influence the removal of micropollutants, as shown for example in Ternes et al. (1999) when comparing the removal efficiencies of estrogens in a German and a Brazilian WWTP.

To sum up, conventional WWTPs have not been designed for micropollutant elimination and have therefore only limited capacity to remove DCF, E2, and EE2. During recent years, various studies have demonstrated this shortcoming and pointed out that treated municipal wastewater even contributes significantly to water pollution (see Table 4). In order to minimize micropollutant discharges into the environment, existing wastewater treatment processes must be upgraded with advanced and alternative methods.

**Table 4:** DCF, E2, and EE2 concentrations in influent and effluents and the removal efficiency by conventional wastewater treatment in Europe since 2002. During secondary treatment, diclofenac had moderate removal rates in different WWTPs in Europe. The removal rates are different, depending on various influences

Compound/WWTP/country	Concentration (μg L^−1^)		Removal efficiency (%)	Reference
	Influent	Effluent		
DCF				
Not described, Germany	3.02^a^	2.51^a^	17	Heberer (2002b)
Conventional WWTP, France, Greece, Italy	–	0.68^a^	–	Andreozzi et al. (2003)
Conventional WWTP, UK	–	0.41–0.46	–	Hilton and Thomas (2004)
4 conventional WWTPs, UK	–	0.599^b^ (0.424^a^)	–	Ashton et al. (2004)
Conventional WWTP, Germany	2.3^b^	1.6^b^	30	Quintana and Reemtsma (2004)
3 conventional WWTPs (1–3) with preliminary clarification	WWTP1 (3 samplings): 3.19–4.11	WWTP1 (3 samplings): 1.53–1.68	WWTP1 (3 samplings): 47–62	Clara et al. (2005)
2 aeration tanks, final clarification, Austria	WWTP2: 1.40	WWTP2: 1.30	WWTP2: 7	
	WWTP3: 0.90	WWTP3: 0.78	WWTP3: 14	
Conventional WWTP, Sweden	0.16	0.12	25	Bendz et al. (2005)
Pilot-scale membrane bioreactor (in 3 sampling periods	3.19–4.11^c^	2.03–3.46^c^	−6.6^d^ to 50.6	Clara et al. (2005)
Conventional WWTP, pilot-scale membrane or fixed bed reactor, Switzerland	–	–	20–40	Joss et al. (2005)
3 conventional WWTPs in EU with secondary or tertiary treatments	–	–	<5	Reemtsma et al.(2006)
Different conventional WWTP, Spain, Belgium, Germany, and Slovenia	0.021–0.148^c^	0.032–1.42^c^	–	Hernando et al. (2006)
5 conventional WWTPs, Croatia	250^a^	215^a^	14	Gros et al. (2006b)
	–	0.21–0.49^c^	–	Rabiet et al. (2006)
Conventional WWTP, Finland	0.42^a^ (0.46^a^)	0.32^b^ (0.35^a^)	24	Vieno (2007)
Conventional WWTP, Norway	295^a^	259^a^	13	Thomas et al. (2007)
Hospital Ulleval, Norway	784^a^			Thomas et al. (2007)
Hospital Rijkshospitalet, Norway	1550^a^			Thomas et al. (2007)
29 WWTPs, municipal and industrial in Bosnia-Herzegovina, Croatia, Serbia	0.859^b^			Terzić et al. (2008)
Conventional WWTP, Sweden, municipal and hospital wastewater	0.23^b^	0.49^b^	−105	Zorita et al. (2009)
WWTP Cilfynydd, Wales, UK: biological treatment-trickling filter beds	0.07	0.12	−71^d^	Kasprzyk-Hordern et al. (2008)
7 conventional WWTPs, Spain			30–100	Gros et al. (2007)
Conventional WWTP, Greece	0.86–2.17^c^	0.15–1.1^c^ 0.41^b^	–	Samaras et al. (2013)
Conventional WWTP, Spain with influent of wastewater from 4 hospitals and municipal wastewater	0.0670^b^	0.043	38	Santos et al. (2013)
Conventional WWTP, Switzerland	1.197^b^	1.187^b^	9	Margot et al. (2013)
8 conventional WWTPs, Greece	0.28^b^	0.11^b^	70	Kosma et al. (2014)
Conventional WWTP, Spain, industrial/municipal wastewater	0.288	0.309	<1	Collado et al. (2014)
Conventional WWTP, France (a) total nitrification + post denitrification; (b) partial nitrification, no denitrification	(a) 184^b^ (b) 384^b^	(a) 52^b^ (b) 171^b^	(a) 72^b^ (b) 55^b^	Mailler et al. (2014)
		0.049^b^/0.043^a^		Loos et al. (2013)
E2				
Conventional WWTP, Norway	12^a^	<3^a^	75	Thomas et al. (2007)
Hospital Ulleval, Norway	28^a^			Thomas et al. (2007)
Hospital Rijkshospitalet, Norway	41^a^			Thomas et al. (2007)
Conventional WWTP, Europe	25.7^b^ 21.5^a^	1.9^b^ 1.0^a^		Janex-Habibi et al. (2009)
EE2				
Conventional WWTP, Norway	<0.3^a^	<0.3^a^		Thomas et al. (2007)
Hospital Ulleval, Norway	<0.3^a^			Thomas et al. (2007)
Hospital Rijkshospitalet, Norway	<0.3^a^			Thomas et al. (2007)
Conventional WWTP, France	1.6^b^ 1.0^a^	0.9^b^ 0.5^a^		Janex-Habibi et al. (2009)

^a^Median

^b^Mean

^c^Min–max

^d^Increase of the effluent concentration relative to the influent concentration

## Advanced and alternative methods

### Mechanical-physical methods

#### Membrane filtration

Microfiltration (MF) and ultrafiltration (UF) are suitable to decrease the concentrations of pharmaceuticals by improved retention of suspended solids in which the more hydrophobic/neutral pharmaceuticals are adsorbed. Hydrophilic substances which are not adsorbed to sludge cannot be retained by MF and UF because of the pore sizes (MF 100–5000 nm, UF 10–100 nm) (Joss et al. 2005). Nanofiltration (NF) and reverse osmosis (RO) have much tighter structures (NF 1–10 nm and RO 0.1–1 nm). In NF and RO membrane processes, the rejection of organic micropollutants like DCF, E2, and EE2 can generally be achieved by size exclusion/steric hindrance, adsorption onto membrane, and/or charge repulsion (Bellona et al. 2004; Xu et al. 2006). The removal efficiency (Table 5) is dependent on properties of the target compound (e.g., molecular weight (MW), molecular diameter (MWd), p*K*_a_, hydrophilicity/hydrophobicity (log *K*_ow_), and diffusion coefficient) and membrane properties. Key membrane properties affecting rejection are pore size, molecular weight cutoff (MWCO), surface charge (measured as zeta potential), hydrophilicity/hydrophobicity, and surface morphology (measured as surface roughness). Additionally, operation conditions like pH value, ionic strength, hardness, the presence of organic matter, and membrane fouling influence the rejection of organic micropollutants (Bellona et al. 2008; Xu et al. 2006; Schäfer et al. 2011). Membrane operation conditions as well as hydrodynamic conditions, such as feedwater recovery, concentration polarization, and feedwater velocity, have been found to influence the rejection of organic micropollutants. Concentrations in influent and effluents and the removal efficiency by advanced biological methods are given in Table 5.

**Table 5:** Concentrations in influent and effluents and the removal efficiency by advanced biological methods

Treatment process	SRT (days)	Removal efficiency (%)	Reference
Diclofenac			
Full-scale WWTP	14–16	68	Kruglova et al. (2014)
Lab-scale SBR	10–12	90	Ribeiro et al. (2013)
Lab-scale MBR	37	23	Quintana et al. (2005)
Single-house MBR	>100	103	Abegglen et al. (2009)
Lab-scale MBR, synthetic WW, HRT 24 h	70	17.3 (mean)	Tadkaew et al. (2011)
E2			
Lab-scale MBR, synthetic WW, HRT 24 h	70	>99.4	Tadkaew et al. (2011)
EE2			
Single-house MBR	>100	77	Abegglen et al. (2009)
Lab-scale MBR, synthetic WW, HRT 24 h	70	93.5 (mean)	Tadkaew et al. (2011)

*MBR* membrane bioreactor, *HRT* hydraulic retention time, *SBR* sequential bioreactor; *WW* wastewater, *WWTP* wastewater treatment plant

In general, if the MW of an organic compound is larger than MWCO of the membrane, the rejection of the compound can be expected to be very high because of steric and electrostatic exclusion. Especially for compounds with a log *K*_ow_ < 2, rejection is governed by MWd compared to the pore size of the membrane. The pH value has a strong influence on the retention of DCF, since the retention of ion species is higher than that of neutral solutes in nanofiltration (Bellona et al. 2004). At lower pH range, where the acidic pharmaceuticals are neutral, larger molecules gave higher retention, because size is the most important parameter in nanofiltration (Urase and Sato 2007).

Table 6 shows the percentage of rejection determined for DCF, E2, and EE2 by different authors along with the type of membrane applied.

**Table 6:** Rejection of DF, E2, and EE2 by membrane filtration

Compound	Membrane type	Rejection (%)^a^	Reference
Diclofenac	NF	100	Radjenović et al. (2009)
	RO	100	Radjenović et al. (2009)
	NF	60	Röhricht et al. (2009)
	NF	65	Röhricht et al. (2010)
	MBR/RO	95	Sahar et al. (2011)
E2	RO	83	Kimura et al. (2007a, b)
	NF/RO	90	Nghiem et al. (2005)
	NF	>99	Weber et al. (2004)
	NF	>95	Yoon et al. (2007)
	RO/NF	High	Drewes et al. (2005)
	NF	77	Bodzek and Dudziak (2006)
	DCMD	≥99.5	Cartinella et al. (2006)
	NF	100	Koyuncu et al. (2008)
	NF/RF	100	Alturki et al. (2010)
	NF	100	McCallum et al. (2008)
EE2	NF	>99	Weber et al. (2004)
	NF	90	Dudziak and Bodzek (2009)
	NF	60	Yoon et al. (2007)
	NF/RO	99	Alturki et al. (2010)

*NF* nanofiltration, *RO* reverse osmosis, *MBR* membrane bioreactor, *DCMD* direct contact membrane distillation

^a^Under optimal conditions

A study by Nghiem et al. (2005) observed that, in the presence of organic matter, micropollutant retention (e.g., hormones) was favored. A clear pH dependency was also found by these authors. As the pH value decreases in the water matrix, the amount of humic acids adsorbed on the membrane increased, as well as the adsorption of the endocrine substances. Koyuncu et al. (2008) explained this by the formation of macromolecular complexes, resulting from the association of humic acids with the hormones. This leads to an increase of size and may enhance the size exclusion effect and the adsorption of hormones onto membranes (Silva et al. 2012).

Röhricht et al. (2009) investigated two different types of submerged nanofiltration flat sheet modules for the removal of pharmaceuticals from WWTP effluents. It was shown that DCF was retained up to 60 %. At pH 8, DCF (p*K*_a_ value of 4.15) was deprotonated and could be rejected by the negatively charged membrane surface. This was in accordance with the statement pointed out by Nghiem et al. (2005) indicating that speciation of pharmaceuticals may result in significant change in rejection as a function of pH, with much greater retention for ionized, negatively charged molecules. When reverse osmosis was applied after conventional activated sludge-ultrafiltration (CAS)-UF/RO and membrane bioreactor MBR/RO, Sahar et al. (2011) reported relatively similar and high elimination of 95 % for DCF in both processes. Despite the highly effective RO treatment, DCF was found in permeates from both units indicating that RO could not completely eliminate this compound and that the additional process was necessary.

One drawback of NF and RO is membrane fouling which may influence the performance of the process as a whole by causing a noticeable decrease in the rejection of organic micropollutants (Ng and Elimelech 2004).

Special types of membrane filtration are direct contact membrane distillation (DCMD) and forward osmosis (FO) which were investigated by Cartinella et al. (2006) for the rejection of hormones. With these techniques, high rejection of over 99.5 % was observed.

The overall conclusion is that membrane filtration is promising, but has not yet been established to provide stable and complete operation at technical scale. There is still great need for research.

#### Adsorption onto sorption materials

Over the years, adsorption has been considered one of the most effective methods to eliminate pollutants from contaminated water (Table 7). Adsorption elimination is based on the uptake of pollutants from the aqueous phase onto a solid phase (sorbent). The affinity of a target compound for its sorbent is often quantified by the specific sorption coefficient, representing the ratio of sorbed and dissolved concentrations of a target compound in equilibrium (Silva et al. 2012). Especially activated carbon (AC) is a well-studied sorbent. In Europe, the most commonly applied ACs are powdered activated carbon (PAC, 5–50 μm diameters) and granular activated carbon (GAC, 100–2400 μm diameters). Table 7 lists different studies concerning the removal of DCF, E2, and EE2 from aqueous solution and WWTP effluents. Zhang et al. (2007) reported that the adsorption process onto AC is strongly influenced by environmental conditions. Contact time has a major effect on removal efficiency. Short contact is likely to lead to significantly lowered adsorption efficiency (Luo et al. 2014). Kumar and Mohan (2011) demonstrated that the adsorption capacity of WWTP effluents is maximum at neutral conditions and at temperatures of up to 30 °C. Sorption of micropollutants onto AC may be reduced by the amount of organic matter and other substances, which are also present in the water matrix because they compete for AC adsorption sites (Fukuhara et al. 2006; Kumar and Mohan 2011; Snyder et al. 2007; Zhang and Zhou 2005). Grover et al. (2011) showed removal efficiencies for DCF, EE2, and E2 of >98 % in a full-scale granular activated carbon plant treating WWTP effluent. The efficiency of GAC-based removal will decrease over time due to saturation of adsorption sites. Therefore, reactors based on GAC have to be operated with care (Luo et al. 2014).

**Table 7:** Advanced technologies

Sorbent	Amount of sorbent	Removal details	Reference
Diclofenac			
AC	30 mg L^−1^	Activated carbon, P110 Hydraffin, (ultrapure water), tubular glass reactor (300 mm long and 50 mm, 93 % after 20 min	Beltrán et al. (2009)
PAC	50 mg L^−1^	Pilot scale, natural water with organic matter spiked with 0.1 μg L^−1^, contact time 4 h, 38–46 %	Snyder et al. (2007)
PAC	10–20 mg L^−1^	300 mg L^−1^ DCF, surface water, 2 h; 76.7 %	Dai et al. (2011)
PAC	23 mg L^−1^ PAC	8, 23, 43 mg L^−1^ in MBR effluent, hospital wastewater, 96, 98, 99 %	Kovalova et al. (2012)
PAC/UF	10–20 mg L^−1^	1.13 μg L^−1^ ± 0.39 WWTP effluent, 10–20 mg L^−1^ PAC, 69 %	Margot et al. (2013)
PAC	5–10 mg L^−1^	WWTP effluent; HRT 25–30 min, pilot scale, up to 98 %	Mailler et al. (2014)
GAC	Packed	Full scale; >98 %	Grover et al. (2011)
GAC	Packed	Full scale (empty bed), 15 min contact	Yang et al. (2011)
GAC/activated sludge	0.5 g L^−1^	Addition of GAC to bioreactor, 93 %	Serrano et al. (2010)
PAC/MBR	1 g L^−1^	Addition of PAC to bioreactor, 93 %	Serrano et al. (2011)
MIP	10 mg L^−1^	300 mg L^−1^ DCF in surface water, MIP 97.6 %	Dai et al. (2011)
E2			
GAC	Packed	Max. adsorption constant: *K*_d_ 12,200 mL g^−1^ with 24.8 μg L^−1^ E2 in water; *K*_d_ 7988 mL g^−1^ with 24.8 μg L^−1^ E2 in WWTP effluent	Zhang and Zhou (2005)
AC	0.03-1.5 mg L^−1^	Various pore size distributions; max. adsorption capacity: 67.6 mg g^−1^ at 1 μg L^−1^ in pure water	Fukuhara et al. (2006)
GAC	Packed	Full scale; 100 %	Grover et al. (2011)
GAC, PAC	Packed, 5 mg L^−1^	Full scale; >90 % for both materials	Snyder et al. (2007)
MIP		25 %	Meng et al. (2005)
MIP	Packed	95 % from 2 μg L^−1^ in deionized water	Le Noir et al. (2007)
MIP	0.5–20 g L^−1^	Dest water, 0.1–1 mg L^−1^ E2, 97 %, 15 mg/g	Lai et al. (2010)
MIP	0.25 g L^−1^	90 % after 2 min incubation, 96 % after long equilibrium	DeMaleki et al. (2010)
EE2			
AC	Packed	Highest adsorption at neutral conditions (95 %), 50 μg L^−1^ EE2 solution (dest water)	Kumar and Mohan (2011)
GAC	Packed	Full scale; 100 %	Grover et al. (2011)
Single-walled CNT		95–98 %, in sea water and brackish water	Joseph et al. (2011)
Multiwalled CNT		25, 50, 75 μg L^−1^ aqueous solution; sorption capacity: 5.6 μg g^−1^	Kumar and Mohan (2012)

*AC* activated carbon, *PAC* powdered activated carbon, *UF* ultrafiltration, *GAC* granular activated carbon, *MIP* molecularly imprinted polymer, *CNT* carbon nanotubes

On the technical scale, PAC is added to WWTP either directly into the activated sludge process or in a subsequent process and needs to be separated from the treated wastewater after application. This is achieved by sedimentation under the addition of flocculation agents or ultrafiltration or sand filtration (Margot et al. 2013). These authors reported mean removal efficiency with GAC/UF combination of 69 %. However, PAC adsorption, with a dosage of 10–20 mg L^−1^, has been proposed as a more efficient alternative compared to GAC treatment (Boehler et al. 2012; Nowotny et al. 2007; Serrano et al. 2011).

The main advantage of using AC to remove micropollutants is that it does not generate toxic or pharmacologically active products (Rivera-Utrilla et al. 2013). The addition of PAC or GAC could also enhance the removal efficiency of micropollutants during biological treatment. Serrano et al. (2010, 2011) reported a significant improvement of DCF removal by adding 1 mg L^−1^ PAC to an MBR treating municipal wastewater and of 0.5 mg L^−1^ GAC to a conventional activated sludge treatment.

Apart from AC, several other sorbent materials have been studied to remove DCF, E2, and EE2. Zhang and Zhou (2005) used chitin, chitosan, an ion-exchange resin, and a waste-derived carbonaceous adsorbent for the removal of E2, but the sorption capacity was lower than with GAC. Another studied sorbent is steroid-based imprinted polymer (molecularly imprinted polymer, MIP). Different groups studied the adsorption of E2 and EE2 onto MIP but only in aqueous solution and never in WWTP effluent. Joseph et al. (2011) reported good removal efficiency up to 98 % from sea water and brackish water with single-walled carbon nanotubes (CNTs). They recorded that removal efficiency is independent of pH and ionic strength. However, increasing concentrations of copresent organic matter decreases the removal of EE2 by 5–15 %.

Overall, the adsorption on activated carbon (PAC and GAC) is a very promising method to reduce trace organic micropollutants from WWTP effluents. Adsorption onto activated carbon is one of the two main technologies that have been identified in Switzerland and Germany with a potential for large-scale application concerning efficiency, energy requirements, and costs (Barjenbruch et al. 2014; Stamm et al. 2015). However, the increased amount of sludge (or loaded activated carbon) for disposal and the high operating costs must not be disregarded.

#### Coagulation-flocculation

In general, the coagulation-flocculation process is applied in WWTP to remove particulate matter. For the elimination of micropollutants, it is inefficient (Matamoros and Salvadó 2013). DCF was removed at a rate of 21.6 % when using FeCl_3_/Al_2_(SO_4_)_3_ as coagulant in hospital wastewater (Suarez et al. 2009). Dissolved humic acids could enhance its elimination (Vieno et al. 2006). The efficiency of coagulation-flocculation can be influenced by different operating conditions such as pH, temperature, alkalinity, presence of divalent cations, and concentration of destabilizing anions (Alexander et al. 2012).

### Physicochemical processes

#### Photolysis

Irradiation with ultraviolet light (UV) is widely used in WWTPs for effluent disinfection prior to discharge into surface water. UV treatment is also known to transform some micropollutants through light absorption on photoactive groups, e.g., photoactive phenolics (Coleman et al. 2004). Two types of photocatalysis are known: (a) direct photolysis via direct absorption of light (Rosenfeldt and Linden 2004a, b) and (b) indirect photolysis, when photosensitizers (dissolved organic matter) adsorb the light and generate reactive oxygenated radicals performing the degradation of the target substance (Caupos et al. 2011). Numerous studies describe degradation of DCF, E2, and EE2 in deionized water but also in WWTP effluents up to 100 % due to their high absorption values (Caupos et al. 2011; Rosenfeldt and Linden 2004a, b; Chowdhury et al. 2010; Silva et al. 2012; De la Cruz et al. 2012).

Kolarova et al. (2013) reported that the removal of DCF in UV_254 nm_ increases with increasing UV dose. While DCF was eliminated only 47 % at 800 J m^−2^, over 98 % removal was observed at 7200 J m^−2^.

Phototransformation has been identified as the important elimination process of DCF in the open environment (Pal et al. 2010). Although the turbidity of wastewater blocks some sunlight, water in the top layers (e.g., in clarifiers) will be well exposed to sunlight irradiation, especially in summer. Therefore, DCF phototransformation will occur in bright sunlight with half-life of less than 1 h. Natural sunlight has also been shown to degrade EE2 (Pal et al. 2010).

#### Radiation

Ionizing radiation such as e-beam accelerators (β-rays) and gamma irradiation (γ-rays, ^60^Co), originally intended for disinfection, is under research for micropollutant degradation. Table 8 lists the facilities in Europe performing wastewater treatment by ionizing radiation.

**Table 8:** Major facilities for wastewater treatment by ionizing radiation (Borrely et al. 1998)

Country	Radiation source	Energy (MeV)	Power (kW)/activity (kCi)	Purpose	Dose (kGy)
Austria	EBA	0.5	12.5	TCE, PCE removal	0.2–2.0
Germany	^60^Co	1.25	135	Disinfection of sludge	2.0–3.0

*EBA* electron beam accelerator, *TCE* trichloroethylene, *PCE* perchloroethylene

The basic differences between these two sources are the dose rate and penetration. Gamma rays are highly penetrating, enabling the processing of bulk material. Ionizing radiation leads to OH radical formation in water dependent on dose, rate, and irradiation time (Borrely et al. 1998; Pikaev 2000; Getoff 2002). When wastewater is irradiated, organic molecules are oxidized. Irradiation excites water electronically and some ions, excited molecules, and free radicals are formed. In the presence of oxygen in water, H^⋅^-atoms and e^−^_aq_ (solvated electrons) are converted into oxidizing species: Perhydroxyl radicals (HO_2_) and anions (O_2_^−^), (HO_2_) and (O_2_^−^) together with OH-radicals initiate degradation of pollutants.

The gamma irradiation (^60^Co) dose required for the elimination of estrogen activity below 1 ng L^−1^ has been found to be about 0.2 kGy (Kimura et al. 2007a). Complete decomposition of DCF (50 mg L^−1^) in aqueous solutions requires 4.0 kGy (^60^Co); however, saturation with N_2_O decreases the dose to 1.0 kGy (Trojanowicz et al. 2012). The sterilization dose for DCF sodium salt, as a pharmaceutical raw material, has been found to be 12.4 kGy (^60^Co) (Ozer et al. 2013). Homlok et al. 2011 described complete removal of DCF with 1.0 kGy. When cost is an issue, it is difficult to give a precise price for irradiation systems in advance because of the many factors involved: the kind and amount of pollutants in water, their properties (chemical, biological, etc.), dose-rate to be used, presence of ozone, combined methods of radiation, and conventional techniques. In general, costs decrease with increase of treatment capacity, and it is possible to say that γ-irradiation costs about four times more than e-beam irradiation because of the high cost of ^60^Co source and the facility (Borrely et al. 1998).

#### Ultrasonic treatment

Ultrasonic treatment is also described as a method to degrade organic micropollutants. Ultrasonic treatment creates three zones of reaction solution: cavitation bubbles, supercritical interface, and bulk solution (Méndez-Arriaga et al. 2008; Naddeo et al. 2010). Méndez-Arriaga et al. (2008) and Chiha et al. (2010) reported that hydrophilic and nonvolatile compounds were mainly degraded in the bulk solution, whereas hydrophobic, nonpolar, and/or volatile compounds react in all three zones. DCF, EE2, and E2 are mainly attacked in bulk solution (Naddeo et al. 2009, 2010; Güyer and Ince 2011). It was found that DCF conversion is enhanced at increased applied power densities, acidic conditions, and in the presence of dissolved air (formation of hydroxyl radicals during ultrasonic treatment). They also reported that biodegradability increased after ultrasonic treatment (Naddeo et al. 2010; Güyer and Ince 2011).

#### Oxidation with single strong oxidation agent

Treatment of WWTP effluents with ozone (O_3_) as oxidizer is one of the most studied chemical treatment technologies in Europe. Ozone oxidizes micropollutants directly or indirectly via HO radical formation (Gerrity et al. 2011). One of the first studies to remove DCF from wastewater was by Ternes et al. (2003). The authors employed ozone concentrations of 5.0 to 15.0 mg L^−1^ to investigate the removal efficiency in WWTP effluents which was >96 %. Magdeburg et al. (2014) described an oxidation efficiency of >90 % for nine different micropollutants including DCF by ozonation of secondary effluent of WWTP using an ozone dose of 0.7 g g^−1^ DOC. These removal efficiencies are in the same range as reported by Hollender et al. (2009), Ternes et al. (2003), and Antoniou et al. (2013). Huber et al. (2005a) investigated the removal of estrogen activity by ozone at three different pH values (3, 7, and 11). Estrogenic activity had disappeared at pH 3, but residual activity remained after oxidation at pH 7 and 11, probably due to by-product formation. Suspended sludge particles could lead to higher ozone consumption, which might reduce the efficiency of ozone for DCF, E2, and EE2 (Hernández-Leal et al. 2011). Recently, Antoniou et al. (2013) investigated the required ozone doses for removing pharmaceuticals in wastewater effluents. They normalized the specific ozone dose to the dissolved organic carbon (DOC) of the effluent, which resulted in an applied ozone dose (DDO_3_/DOC) ratio of 0.67 for DCF.

Because ozonation has been considered a second promising technology in Europe during the last years, some of WWTPs in Switzerland and Germany have been upgraded with ozone oxidation or/and activated carbon adsorption (Barjenbruch et al. 2014; Stamm et al. 2015). While in an adsorptive process using PAC or GAC organic micropollutants are removed, oxidation processes like ozonation do not result in complete mineralization of micropollutants but in the formation of predominantly unknown transformation products (TP) with unknown toxicity. Additionally, inorganic by-product from oxidation will be formed (Joss et al. 2008; Stadler et al. 2012).

In general, the TP have low concentrations as well as insignificant estrogenic and antimicrobial activities compared to the parent compound (Hollender et al. 2009; Reungoat et al. 2011). To further reduce TP, biological post-filtration over activated carbon or sand can be considered (Luo et al. 2014).

Huber et al. (2005b) investigated the potential of chlorine dioxide (ClO_2_) for the oxidation of DCF and EE2 during water treatment (drinking water, groundwater, and lake water; not wastewater). ClO_2_ is a stable free radical that reacts with micropollutants through a one-electron transfer and is a highly selective oxidant with respect to specific functional groups like phenolic groups (Huber et al. 2005b). DCF (1 μg L^−1^) was readily oxidized with ClO_2_ in 30 min with a dose of 0.95–11.5 mg L^−1^ ClO_2_ but in lake water only after 60 min. EE2 (11 μg L^−1^) reacted very fast in less than 5 min with 0.1 mg L^−1^ ClO_2_ in groundwater (Huber et al. 2005b). There are no studies available for WWTP effluents.

When the degradation of EE2 and DCF was studied under MnO_2_ or biogenic produced manganese oxides (BioMnOx) in a synthetic wastewater (Forrez et al. 2009, 2010), removal of up to 80 % could be verified. At neutral pH, the diclofenac oxidation with BioMnOx was 10-fold faster than with chemically produced MnO_2_. The main advantage of BioMnOx over chemical MnO_2_ is the ability of bacteria to reoxidize the formed Mn^2+^, which inhibits the oxidation of DCF. Diclofenac oxidation was proportional to the amount of BioMnOx dosed, and the pseudo first-order rate constant *k* was 6-fold higher when pH was decreased from 6.8 to 6.2. These results combined with previous studies suggest the potential of BioMnOx for WWTP effluent polishing, but the technique is not yet used in technical scale.

#### Advanced oxidation processes

Advanced oxidation processes (AOPs) are very effective in the oxidation of numerous organic and inorganic pollutants. AOPs are based on the generation of free radicals, mainly the HO^⋅^ radical, with high oxidizing power, which can successfully attack most organic molecules with elevated reaction constants from 10^6^ to 10^9^ M^−1^ s^−1^ (Von Sonntag 2008; Huber et al. 2003; Rivera-Utrilla et al. 2013). This makes AOPs superior to treat organic molecules with high chemical stability and/or low biodegradability (Oller et al. 2011). Due to their electrophilic nature, HO^⋅^ radicals oxidize almost all electron-rich organic substances, eventually converting them to carbon dioxide and water. Most AOPs use a combination of two different oxidants (e.g., O_3_/H_2_O_2_), oxidant and irradiation (e.g., H_2_O_2_/UV), oxidant and catalyst (e.g., H_2_O_2_/Fe^2+/3+^ (Fenton)), oxidant and photocatalyst (e.g., H_2_O_2_/UV/Fe^2+/3+^ (photo-Fenton), or oxidant and ultrasonic (e.g., H_2_O_2_/ultrasonic) (Von Gunten 2003; De la Cruz et al. 2012). Many of these advanced systems have been evaluated in laboratory batch tests and have yet to be applied on technical scale; thus, there is a lack of good quality data on the mechanisms involved, the influence of operational variables, the reaction kinetics, and reactor design issues.

Gerrity et al. (2011) reported high removal efficiency for DCF of >99 % and of E2 of >83 % in a pilot-scale treatment plant of WWTP effluent with O_3_/H_2_O_2_. Recently, Rivera-Utrilla et al. (2013) and Silva et al. (2012) exhaustively reviewed the literature on the removal of pharmaceuticals from water, summarizing also the performances of different water treatment systems including advanced technologies. In the case of DCF, EE2, and E2, some promising technologies have been identified and summarized in Table 9.

**Table 9:** Advanced methods and removal efficiency of DCF, E2, and EE2

Method	Initial concentration	Method, removal efficiency	Reference
DCF			
FeCl_3_/Al_2_(SO_4_)_3_	14–18 μg L^−1^ (municipal wastewater) 10–18 μg L^−1^ 10–18 μg L^−1^	Coagulation-flocculation; 70 % FeCl_2_/68 % Al_2_(SO_4_)_3_, with aluminum polychloride; 50 % flotation with low fat wastewater 12 °C, 25 %; 25 °C; 40 % flotation with high fat wastewater 22 °C, 25 %; 25 °C, 48 %	Carballa et al. (2005)
FeCl_3_/Al_2_(SO_4_)_3_	Municipal wastewater	Coagulation-flocculation, 21.6 %(mean)	Suarez et al. (2009)
UV-A	15 mg L^−1^ (deionized water)	50 mL cylindrical quartz glass UV-reactor; photocatalytic treatment 1500 W xenon arc lamp (750 W m^−2^) 100 % in 1 h	Calza et al. (2006)
UV-A	10 mg L^−1^ (deionized water)	350 mL laboratory-scale photoreactor; 9 W UV-A lamp at a fluence 0.69 kWh m^−2^, TiO_2_, 85 % after 240 min	Achilleos et al. (2010)
UV_254 nm_	0.518 μg L^−1^ (WWTP effluent)	10 min, 100 %	De la Cruz et al. (2012)
UV_200–800 nm_	9.24 mg L^−1^ (deionized water)	Low and medium pressure: 97–98 %	Lekkerkerker-Teunissen et al. (2012)
UV_254 nm_	0.858 μg L^−1^ (MBR effluent hospital wastewater)	800, 2400, 7200 J m^−2^; 47 %, 88 %, >98 %	Kovalova et al. (2013)
UV/H_2_O_2_	2.8 mg L^−1^	LP-Hg lamp (2.51 × 10^−6^ E s^−1^) [H_2_O_2_] 5 and 10 mM, pH 7.8, *T* = 298 K; 100 % in 2 min	Andreozzi et al. (2003)
UV/H_2_O_2_	1 mM (296 mg L^−1^) solution with double glass-distilled water	UV/H_2_O_2_ oxidation, 17 W low-pressure mercury monochromatic lamp, annular reactor (0.420 L); complete in 10 min	Vogna et al. (2004)
UV-A/TiO_2_/H_2_O_2_	(Synthetic WWTP effluent)	UV-A: 2.8 × 10^−6^ E s^−1^, [TiO_2_]: 0.1 g L^−1^, [H_2_O_2_]: 100 mg L^−1^; fixed bed reactor	Pablos et al. (2013)
UV_200–800 nm_/H_2_O_2_	9.24 mg L^−1^ (deionized water)	Low and medium pressure, [H_2_O_2_]: 5–10 mg L^−1^, 97–98 %	Lekkerkerker-Teunissen et al. (2012)
UV_254 nm_/H_2_O_2_	0.518 μg L^−1^ (WWTP effluent)	10 min, [H_2_O_2_]: 50 mg L^−1^, 100 %	De la Cruz et al. (2012)
UV_254 nm_/Fenton (photo-Fenton)	0.518 μg L^−1^ (WWTP effluent)	10 min, UV_254 nm_, [Fe^2+^]: 5 mg L^−1^, [H_2_O_2_]: 25–50 mg L^−1^, 100 %	De la Cruz et al. (2012)
UV_254 nm_/H_2_O_2_/Fe UV_254 nm_/H_2_O_2_	0.49–1.3 μg L^−1^ (WWTP effluent) 0.49–1.3 μg L^−1^ (WWTP effluent)	[H_2_O_2_]: 20–30 mg L^−1^, [Fe^2+^]: 2 mg L^−1^: 99–100 % [H_2_O_2_]: 20–30 mg L^−1^, 99–100 %	De la Cruz et al. (2013)
Radiation	0.1–1 mM	0.1–1 mM DCF: few kGy doses sufficient; 0.1 mM DCF—complete degradation with 1 kGy dose	Homlok et al. (2011)
Radiation	50 mg L^−1^	100 % with 4.0 kGy dose (^60^Co), or with 1.0 kGy, when saturated with N_2_O	Trojanowicz et al. (2012)
Radiation	DCF sodium salt	12.4 kGy (^60^Co)	Ozer et al. (2013)
Ultrasonic	2–5 mg L^−1^ (deionized water)	pH (3.5–11), power density (25–100 W L^−1^), TOC removal of 19 % after 60 min	Naddeo et al. (2009)
Ultrasonication	30 μM DCF (deionized water)	pH 3, frequency: 861 kHz, 90 min sonication in the presence of 8.9 mM reactive zero-valent iron (ZVI), 0.01 mM reactive divalent iron (DVI), and 0.001 mM nonreactive iron superoxide nanoparticles (NPI) were 22, 43, and 30 %, respectively	Güyer and Ince (2011)
O_3_	1.3	[O_3_]: 5–10 mg L^−1^, >96 %	Ternes et al. (2003)
O_3_	1 mM (296 mg L^−1^) solution with double glass-distilled water	[O_3_]: 5 mg L^−1^ semibatch glass reactor (1.090 L); almost completely after 10 min	Vogna et al. (2004)
O_3_	10 μg L^−1^	K_O3_ = 6.8 × 10^5^ M^−1^ s^−1^ [O_3_]: 0.016 mg L^−1^, 100 %	Sein et al. (2008)
O_3_	200 mg L^−1^ (Milli-Q water)	Ozonation, 1 L batch reactor; almost completely after 30 min	Coelho et al. (2009)
O_3_	0.015 (WWTP effluent)	Technical scale; [O_3_]: 5 mg L^−1^, >90 % in 15 min	Sui et al. (2010)
O_3_	0.858 μg L^−1^ (MBR effluent hospital wastewater)	[O_3_]: 4.2, 5.8, 7 mg L^−1^; 100 % for all three O_3_ concentrations	Kovalova et al. (2013)
O_3_	1 μg L^−1^ (WWTP effluent)	[O_3_]: 0.5–12.0 mg L^−1^	Antoniou et al. (2013)
O_3_	1.13 μg L^−1^ ± 0.39	5.7 mg L^−1^ ozone dosage, technical scale; WWTP effluent, 94 %	Margot et al. (2013)
O_3_	1 μg L^−1^ (WWTP effluent)	[O_3_]: 0.5–12 mg L^−1^, 100 %	Antoniou et al. (2013)
ClO_2_	1 μg L^−1^ (ground and surface water)	[ClO_2_]: 0.95–11.5 mg L^−1^, 30–60 min, 100 %	Huber et al. (2005b)
O_3_/H_2_O_2_	0.165 (average) WWTP effluent	Pilot scale; [O_3_]: 5 mg L^−1^; [H_2_O_2_]: 3.5 mg L^−1^; >99 %	Gerrity et al. (2011)
O_3_/UV-A/TiO_2_	30 and 80 mg L^−1^ (ultrapure water and WWTP effluent)	Cylindrical borosilicate glass photoreactor (0.45 m height and 0.08 m inside diameter), 100 % within 6 min	Aguinaco et al. (2012)
O_2_/UVA/TiO_2_ O_3_/UVA/TiO_2_	10^−4^ M/L solution in Milli-Q water	Cylindrical borosilicate glass photoreactor (0.45 m height, 0.08 m diameter); ozonation, almost completely after 7 min O_2_/UVA/TiO_2_, 90 % after 10 min O_3_/UVA/TiO_2_, 95 % after 10 min	García-Araya et al. (2010)
Fenton	0.518 μg L^−1^ (WWTP effluent)	30 min, [Fe^2+^]: 5 mg L^−1^, [H_2_O_2_]: 25–50 mg L^−1^, 24 %	De la Cruz et al. (2012)
Sonolysis TiO_2_/sonolysis	50 mg L^−1^ (deionized water)	300 mL batch reactor; sonolysis, 90 % after 60 min; sonolysis, TiO_2_ catalyst, 84 % after 30 min; sonolysis, SiO_2_ catalyst, 80 % after 30 min; sonolysis, TiO_2_ and SiO_2_ catalysts, 80 % after 30 min	Hartmann et al. (2008)
BDD/Si	175 mg L^−1^ (deionized water)	150 mL batch reactor pH 6.5 50 mA cm^−2^: 95.1 % after 360 min 100 mA cm^−2^: 98.9 % after 360 min 300 mA cm^−2^: 100 % after 300 min 450 mA cm^−2^: 100 % after 200 min	Brillas et al. (2010)
BDD/Nb	300 mg L^−1^ (bidistilled water)	Batch reactor 100 mL; [Na_2_SO_4_] = 0.1 surface area electrode: 6 cm; 42 mA cm^−2^; 99.8 % within 600 min	Vedenyapina et al. (2011)
BDD/Ti	150 mg L^−1^	Batch reactor; pH 6.5; current densities = 10, 15, and 20 mA cm^−2^; higher DCF decay achieved at current density of 15 mA cm^−2^. Higher current density leads to oxygen evolution and less efficiency	Coria et al. (2014)
BDD/Nb	50 μM (deionized water, hard tap water, WWTP effluent)	Batch reactor, 3 L, 3.5 A, 100 % after 15 min in deionized water, in 20 min in hard tap water, in 30 min in WWTP effluent	Rajab et al. (2013)
Pulsed corona discharge	5 mg L^−1^ (tap water)	Reactor (solution volume 55 mL); 100 % after 7 min	Dobrin et al. (2013)
Magnetic nanoscaled catalyst cobalt ferrite/oxone	33.77 μM (deionized water)	250 mL glass bottle; 100 % in 15 min	Deng et al. (2003)
PdFe	32 mM (bidistilled water)	Plated elemental iron (PdFe), anoxic condition, batch experiment 80 % within 10 min, 100 % after 2 h	Ghauch et al. (2010)
Fe^0^-based trimetallic system	32 μM (bidistilled water)	Anoxic condition, batch experiment PdNiFe, 100 % after 1 h PdCuFe, 80 % after 1 h NiPdFe, 80 % after 1 h	Ghauch et al. (2011)
E2			
O_3_	0.5–5 μg L^−1^ (WWTP effluent)	[O_3_]: ≥2 mg L^−1^, 90–99 %	Huber et al. (2005a)
UV	5 μM (deionized water)	LP-UV, MP-UV, reduction of estrogenic activity lower relevant concentrations	Rosenfeldt et al. (2006)
UV/H_2_O_2_	5 μM (deionized water)	LP-UV + 5 mg L^−1^ H_2_O_2_; >90 % MP-UV + 5 mg L^−1^ H_2_O_2_; >90 %	Rosenfeldt et al. (2007)
UV-A/TiO_2_	500 μg L^−1^ (deionized water)	[TiO_2_]: 10 mg L^−1^ Degradation efficiency increases with increasing pH value	Karpova et al. (2007)
UV-A/TiO_2_	10 μg L^−1^ (distilled water)	55 min for 100 %, 24 min for 90 %	Coleman et al. (2004)
O_3_/H_2_O_2_	0.003 (average) WWTP effluent	Pilot scale; [O_3_]: 5 mg L^−1^; [H_2_O_2_]: 3.5 mg L^−1^; >83 %	Gerrity et al. (2011)
BDD/Si	500 μg L^−1^ (distilled water)	500 mL batch reactor pH 6 12.5 mA cm^−2^: 100 % after 40 min 25 mA/cm^−2^: 100 % after 40 min	Murugananthan et al. (2007)
EE2			
O_3_	4 μmol/L (natural water)	[O_3_]: 1.5–7.5 μmol L^−1^, removal strongly depends on pH value	Huber et al. (2003)
O_3_	0.5–5 μg L^−1^ (WWTP effluent)	[O_3_]: ≥2 mg L^−1^, 90–99 %	Huber et al. (2005a)
ClO_2_	1 μg L^−1^ (groundwater)	[ClO_2_]: 0.1 mg L^−1^, <5 min, 100 %	Huber et al. (2005b)
MnO_2_	5 mg L^−1^ day^−1^ 40 mg L^−1^ day^−1^	93 % 75 %	Forrez et al. (2009)
Biologically produced MnO_2_	40 mg L^−1^ day^−1^	57 %	Forrez et al. (2009)
UV-A/TiO_2_	10 μg L^−1^ (distilled water)	50 min for 100 %, 27.5 min for 90 %	Coleman et al. (2004)
Ultrasonic/O_3_		Ultrasonic ozonation (US/O_3_) and photocatalytic ozonation (PC/O_3_) under different conditions involving supplied ozone dose, pH value and humic acid (HA) concentration of the effluent, ultrasonic radiation power, and photocatalyst dose; <13.3 % removal rate for EE2	Zhou et al. (2015)

In recent years, the electrochemical based AOPs (EAOPs) have gained more attention due to several advantages over normal AOPs (Martínez-Huitle and Ferro 2006; Sirés et al. 2014; Sirés and Brillas 2012). The EAOPs are clean technologies that do not use any chemicals during the process. Besides, the operation under mild (room temperature and ambient pressure) and versatile (applicable to CODs of 0.1 to 100 g L^−1^) conditions, the high energy efficiency, and the easy handling are—among others—advantages that distinguish the application of EAOPs from classical methods (Sirés et al. 2014). The EAOPs can be classified into two groups: (1) anodic oxidation (AO), where, at the anode surface, in situ OH radical is generated (e.g., boron-doped diamond electrodes (BDD); and (2) electro-Fenton (EF), via in situ electrocatalytically generated Fenton’s reagent, including different coupling with other photo-, sono-, or physio-chemical treatment methods (Oturan and Aaron 2014). The anode material is a crucial element in an EAOP. Originally, the AO process was conducted with high O_2_ evolution overpotential anodes (Brillas and Martinez-Huitle 2011), such as Pt, graphite, PbO_2_, doped SnO_2_, IrO_2_, or dimensionally stable (DSA) anodes. An essential feature of the anode material is to inhibit the generation of oxygen molecules and to impose the formation of significant amounts of oxidizing agent such as hydroxyl radicals (Comninellis et al. 2008). The previously reported electrode materials are not stable against the reactive species formed on their surfaces and erosion of the material would be possible (Barrera-Díaz et al. 2014). The boron-doped diamond (BDD) electrode, however, shows an outstanding specificity for electrochemical oxidation processes promoting it as a very promising anode material (Fryda et al. 2003; Kraft et al. 2003; Tröster et al. 2004; Martínez-Huitle and Quiroz Alfaro 2008). Depending on the source of the water, the degradation progress may be quite heterogeneous, due to competitive reactions with organic and inorganic matter at high concentrations in hard water and WWTP effluents (Wert et al. 2011; Rajab et al. 2013). AOPs and EAOPs have been tested mainly in lab scale and are far from technical application, also because of by-product formation and costs.

Heterogeneous photocatalytic oxidation is a method relying on the capability of photocatalysts like titanium dioxide (TiO_2_), zinc oxide (ZnO), zinc sulfide (ZnS), ferric oxide (Fe_2_O_3_), silicon (si), and tin oxide (SnO_2_) to act as sensitizers for light-induced redox processes (Silva et al. 2012). TiO_2_ is the most widely cited photocatalyst due to its considerable activity, high stability, nonenvironmental impact, and low cost (Augugliaro et al. 2012; Silva et al. 2012). The heterogeneous photocatalysis process using TiO_2_ was applied successfully for the removal of DCF, E2, and EE2 with high removal efficiencies in aqueous solutions including WWTP effluents. Selected studies are listed in Table 9. Coleman et al. (2004) found a selectivity for estrogens EE2 > E2. The removal efficiency of E2 and EE2 increases with increasing pH value (Karpova et al. 2007).

None of the AOPs mentioned above will result in a complete mineralization of organic micropollutants. Transformation products are formed which could be biodegradable but also toxic, bound, or mobile, which makes a biological posttreatment to degrade these TP indispensable (Christensen et al. 2009).

### Phytoremediation

#### DCF

Recent reviews have indicated that besides longer SRT and HRT, the implementation of wetland plants might improve the performance of older WWTPs in small settlements. Recommendations have been made to add lagunar phytoremediation modules to improve the removal of PPCPs even more effectively (Schröder et al. 2007). In such systems, the uptake and removal of DCF and estrogens relies on the biology of green plants and their accompanying rhizospheric microbial communities, in analogy to mammalian detoxification systems.

**Fig. 2:**
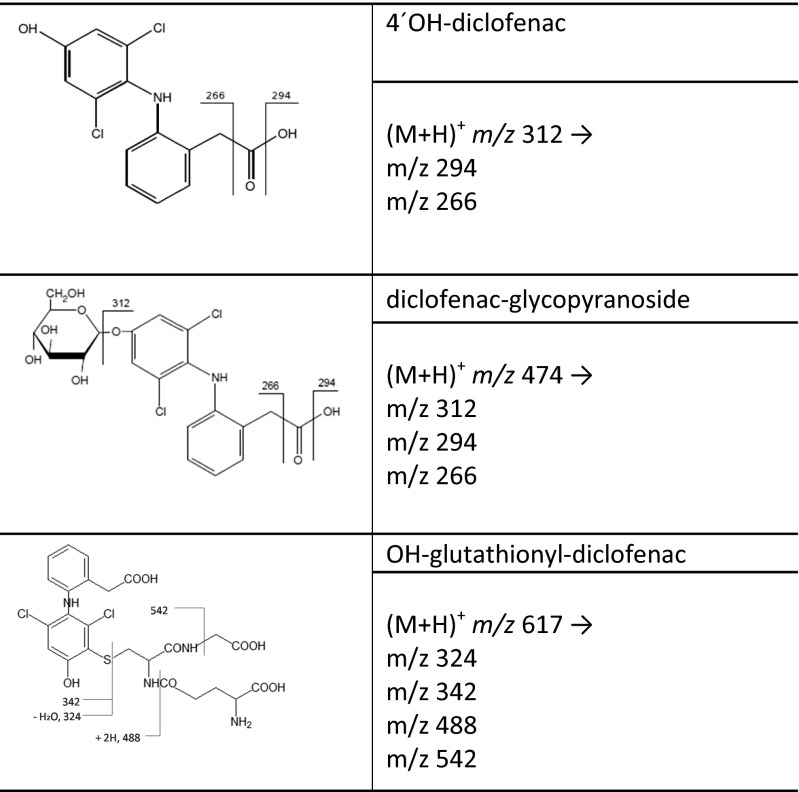
Chemical structures of diclofenac metabolites identified in plants and the characteristic mass transitions obtained in positive ionization mode by LC-MS/MS analysis

In humans, many drugs undergo a cascade of different reactions. An initial activation reaction is frequently followed by conjugation with smaller biomolecules like glucuronic acid or sulfuric acid. These modifications of the parent drug increase its solubility and the potential for excretion of active metabolites. The very same mechanisms exist in plants (Schröder and Collins 2002), and it has been demonstrated that they are active against a broad spectrum of xenobiotic compounds. In mechanistic laboratory and greenhouse studies with different plant species (*Armoracia rusticana*, *Brassica juncea*, *Hordeum vulgare*, *Lupinus luteolus*, *Typha latifolia*, *Phragmites australis*), the uptake and subsequent detoxification of DCF has recently been demonstrated (Kotyza et al. 2010; Huber et al. 2012; Bartha et al. 2014). Since DCF is a weak acid, its uptake in the plant with the transpiration stream is not inhibited, and significant concentrations accumulate in both roots and shoots of investigated species. Interestingly, the pharmaceutical is attacked by enzymes very similar to mammalian ones. After activation by P450 or peroxidase enzymes, the hydroxylated primary metabolites were conjugated either with glucose, or glutathione (Fig. 2), rendering the products more water soluble and nontoxic.

#### Estradiols

Phytoremediation of ECDs has been investigated in different studies. The removal of 17 β-estradiol and 17 α-ethinylestradiol from contaminated waters by macrophytes was discussed by Trueman and Erber (2013). The authors studied the uptake of two estrogenic compounds as well as Bisphenol A into the tissues of two *Potamogeton* species. Whereas the amounts of the estradiol compounds in both species were rather low (15.7 ng L^−1^) compared to the concentration in the water, the plants took up a considerable amount of Bisphenol A (8.3 μg g^−1^ DW).

The use of vertical flow wetlands is a common technique in phytoremediation. Planted with common reed (*Phragmites australis*) these systems have been tested for the removal of endocrine disruptors from wastewaters (Song et al. 2009). The authors reported a maximal removal efficiency of 67.8 ± 28.0 %, 84.0 ± 15.4 % and 75.3 ± 17.6 % for E1, E2 and EE2, respectively. In a comparison of different wetland depths, they found the shallowest (7.5 cm) to be the most efficient one to remove EDCs from the water body.

Apart from macrophytes, Duckweed (*Lemna* species) and a mixture of algae and cyanobacteria were studied for their capacity to remove ECDs from synthetic wastewater under different conditions in batch experiments. In the presence of duckweed and algae, effective removal of the estrogens E1, E2, and EE2 from waters was observed, even at nanogram per liter concentrations (Shi et al. 2010). The accelerated removal of estrogens is probably due to its absorption on the duckweed or algae and subsequent degradation by microorganisms adhering to the plants. However, plant metabolism was not excluded, and duckweed showed a slightly higher efficiency to remove estrogens than algae.

Generally, for the use of plants in any remediation scenario, the selection of the most suitable species to do the job is crucial (Schröder 2007). This includes knowledge of the plants’ metabolic capacity, their ability to grow under given environmental conditions, and favorable milieu (e.g., oxygen, root surface, chemical milieu) for plant-associated microorganisms which may contribute to degradation and removal of the pollutants in manifold ways. When using macrophyte species, it remains important to remove all plants after remediation to avoid the release of sequestered nutrients and pollutants back into the system during decomposition. In many cases, especially for small settlements, phytoremediation, when properly performed, may be an appropriate and cost-effective way to remove considerable amounts of pollutants from aquatic ecosystems and WWTPs.

## Ecotoxicology and risk assessment

While compilations on the occurrence and fate of pharmaceutically active compounds and their metabolites in sewage and potable water are increasingly available and point to the danger of their widespread distribution (Sweetman 2002; Petrović et al. 2014; Škrbić et al. 2014), the environmental effects of their presence alone and in mixtures have so far not been properly addressed (Halling-Sorensen et al. 1998; Daughton 2001; Ternes 2001; Arnold et al. 2013; Manickum and John 2014; Vieno and Sillanpää 2014; Shore et al. 2014; Gabet-Giraud et al. 2014). In this section, the main ecotoxicological issues related to diclofenac and EE2 are summarized; for a wider discussion on the topic, the reader is referred to the accompanying paper on ecotoxicity of micropollutants (Papa et al., in preparation).

### Pure compound approach

The objective of an environmental risk assessment (ERA) is to prove, beyond reasonable doubt, that the compounds are safe for all manmade and natural ecosystems which they may enter, such as WWTPs, rivers, and soil. A compound is judged as having little or no environmental risk if the predicted environmental concentration (PECs)—which is the concentration of the compound expected to be found in the environment—is lower than the predicted no effect concentration (PNEC)—that is, the concentration that causes no adverse effect to the environment. However, the compounds are rarely present alone in the environment; hence, the concentrations of compounds that are asserting similar adverse effects in the environment are usually added for the ERA (Fent et al. 2006). Moreover, since many compounds may be altered prior to or during treatment, and/or in the receiving organisms themselves, further potential metabolites (relevant for many pharmaceuticals) and transformation products should be included in the assessment.

The ERA is a tiered process that progresses from using screening-level tests and conservative assumptions to increasingly more realistic assumptions (EC 2003). The PNEC is typically obtained from the lowest effect concentration (LOEC) for the most sensitive species. However, ecotoxicity data are often limited or not available, especially for metabolites and transformation products. Hence, the traditional ERA, as described by the European Commission Technical Guidance Document (TGD), allows the use of assessment factors to account for the uncertainty in deriving PNEC values based on acute toxicity data and a limited number of species (EC 2003). For biologically active compounds such as pharmaceuticals, this approach may overlook sublethal and subtle subcellular effects that may occur in some species at much lower concentrations during chronic exposure (Fent et al. 2006).

Typical PNEC values for diclofenac and EE2 when derived from traditional ERA using acute toxicity data lies in the milligram per liter range, while chronic histopathological effects have been observed in rainbow trout after 28 days of exposure to 1–5 μg L^−1^ DCF (Schwaiger et al. 2004; Triebskorn et al. 2004). The fact that diclofenac also bioaccumulates is also of concern and should be addressed properly (Fent et al. 2006). Kallio et al. (2010) found that the total bioconcentration factors (BCF_total_) for diclofenac and its metabolites in rainbow trout bile varied between individuals and was roughly estimated to range from 320 to 950.

As for EE2, Caldwell et al. (2008, 2012) proposed a PNEC value of 0.1 ng L^−1^ in surface water. It was derived from a species sensitivity distribution using no observed effect concentrations (NOECs) for reproductive effects from 42 papers in 26 species and was determined as the median hazardous concentration at which 5 % of the species tested were affected (HC5,50).

#### Whole-effects approach

Another approach to the assessment of micropollutants would be to switch from a compounds-oriented to an effects-oriented one, in order to take into account (i) unknown/undetected compounds, like metabolites and parent compounds, and (ii) the mixture effects of substances, either synergistic or antagonistic. Therefore, comprehensive bioanalytical tools can directly measure the specific biological activity of groups of chemicals. This is just the case for EE2: indeed, when assessing its ecotoxicological effects, the main threat is represented by the induced estrogenic activity, i.e., a specific mode of toxic action directly related to all those molecules (then called endocrine-disrupting compounds, EDCs) that can mimic, block, or interfere with hormonal activities in living organisms. In regard to ERA for the receiving water bodies, the main adverse impact related to this kind of biological activity is represented by impaired reproductive performance in wildlife and especially in fish: levels of 0.1–0.4 ng L^−1^ were postulated by Jarošová et al. (2014) for safe concentrations of estrogenic equivalents (EEQs) for municipal WWTP effluents. The concept of estrogenic equivalents is used to group all the chemicals able to induce this specific mode of toxic action, and is measured via estrogenic activity assays (Leusch et al. 2010). They are based on the interaction between compounds and estrogenic receptors and can be performed with cells (E-SCREEN, ER-CALUX, MELN, and KBluc assays) and yeast (YES assay) (Leusch et al. 2010).

## Modeling of diclofenac and hormones

In wastewater treatment plants, mathematical models are routinely used for plant design, optimization, and control. In general, the most commonly used models are derived from the activated sludge models (ASMs) that were developed to predict the degradation of organic carbon, nitrogen, and phosphorus (Henze et al. 2000). In recent years, ASMs have been extended to include the degradation of micropollutants, including pharmaceutical compounds such as diclofenac and estrogens such as E2 and EE2 (Lust et al. 2012; Plosz et al. 2012). These models have been developed to primarily include removal mechanisms associated with biotransformation and adsorption, since removal via volatilization/stripping has been found to be comparatively negligible for these compounds.

In the modeling of biotransformation processes, separate kinetic expressions are typically employed in order to describe both aerobic and anoxic degradation (Joss et al. 2004, 2006). Biotransformation, described by Joss et al. (2004, 2006) through pseudo first-order degradation kinetics, generally occurs at a higher rate aerobically than anoxically, due to the contribution of autotrophic bacteria (i.e., nitrifiers), which often display higher kinetics for pharmaceutical degradation than heterotrophic bacteria. Adsorption and desorption are typically estimated assuming an equilibrium between the dissolved and sorbed concentration of the respective pharmaceutical. This equilibrium is dependent on the suspended solids concentration. The sorption behavior for pharmaceuticals such as DCF and estrogens will routinely be estimated by their *K*_d_ (see “Conventional treatment systems and their shortcomings”).

The ASM-X model developed by Plosz et al. (2012) for DCF (and other pharmaceuticals) incorporates expressions involving both the biotransformation of the micropollutant and its reformation into the parent compound. This is due to the fact that closely related DCF conjugates can also be found in influent wastewaters (typically generated as human metabolites), where the parent DCF molecule is then reliberated biologically through conjugate-cleavage in the activated sludge process. The biodegradation of DCF is predicted through both direct biodegradation as well as through cometabolic biodegradation via other soluble substrates present in the wastewater. While sorption and desorption of DCF to the sludge was predicted through its *K*_d_, Plosz et al. (2012) also employed a term to predict the fraction of DCF sequestered in sludge to account for the fact that the sorbed DCF detected in the activated sludge was substantially higher than that predicted by liquid–solid equilibrium.

Models describing the biodegradation of estrogens (estrone (E1), E2) and EE2 have also been developed (Monteith et al. 2008; Lust et al. 2012), which also predict both their biodegradation and adsorption/desorption to sludge through liquid–solid equilibrium (*K*_d_) coefficients. With respect to biodegradation, since E1 is formed from E2 biodegradation, sequential degradation of E2 to E1 has been considered in these models as the major biochemical pathway. Formation of conjugated estrogens was also incorporated into the model of Lust et al. (2012).

Recently, the ASM-X model has also been incorporated into the benchmark simulation model (BSM) structure in order to facilitate its integration with plant-wide control strategy scenarios (Snip et al. 2014). This study also proposed a dynamic influent prediction tool to estimate the concentration of, e.g., DCF as a function of administration pattern, bioavailability, and residence time in the human body.

Now it will be necessary to include the transformation of these compounds in tertiary treatment processes, such as filtration, UV, and ozonation, eventually combined with phytoremediation, considering the fact that these processes have been typically found to contribute to a substantial portion of the removal of pharmaceutical compounds. Of course, kinetic approaches regarding the generation of TPs from DCF or estrogen biotransformation or oxidation processes must be included, particularly in view of the toxicity that such metabolites may exhibit, often higher than the parent compounds themselves, and that they may constitute the bulk chemical from reaching and persisting within the environment.

WWTP effluents, if properly treated, can be reclaimed and reused for determined restricted uses, contributing in this way to the reduction of water pollutants and the pressure over the worldwide water scarcity. It will be also up to the models to forecast limits of such technologies and develop action plans for optimized remediation techniques aiming at avoiding the release of these substances into the environment, to preserve the ecosystem but also protect biodiversity.

## Economics

The level of pollutant removal from wastewater exponentially increases the associated costs. When the treatment involves micropollutants like the DCF and estrogens, there is also an additional cost due to the advanced technology required. Owen and Jobling (2012) reported that, in order to remove EE2 from wastewater to comply with the proposed legislation, GAC systems should be implemented in all conventional WWTPs. The investment cost of such a system for a town of 250,000 inhabitants would be around 8 million €, and its operating costs around 800,000 € per year. A similar finding was drawn by Jones et al. (2007), who concluded that the cost of utilizing drinking water technologies to treat wastewater will likely be really expensive. In particular, it was estimated that for medium- and large-sized WWTPs, the capital cost of sand filter and membranes exceeded the cost of the basic activated sludge WWTP by 2.63 £ and 1.5 million £, respectively. Moreover, the potential operating costs of the extra treatment processes would be also significantly higher than standard treatment, since they would increase by around six times.

Since economies of scale apply to wastewater treatment facilities (Tsagarakis et al. 2003; Fraquelli and Giandrone 2003; Hernandez-Sancho et al. 2011), the cost for removing micropollutants from small installations will be extremely high. Advances in technology are promising in achieving high removal rates for micropollutants (see section “Advanced and alternative methods”) but should be achieved with affordable tariffs for the residents. The main challenge is to quantify the benefits of micropollutant removal, which is difficult, since the level of damage to the environment and biodiversity has yet to be fully taken into account (Luo et al. 2014; Vieno and Sillanpää 2014; Pereira et al. 2015).

In such cases, nonmarket valuation can be applied so that a monetary value can be attributed to the benefits derived from the wastewater treatment (Menegaki et al. 2007; Genius et al. 2012). Since costs are transferred to the residents, any new investment for advanced treatment should be investigated for acceptance and willingness to pay for the capital and operation cost. This is essential since residents will be asked to pay for higher tariffs (Genius et al. 2005).

Literature is very scarce on nonmarket valuation of micropollutants. In a contingent valuation study, Kotchen et al. (2009) investigated the willingness to pay for a surcharge on prescriptions to support a pharmaceutical disposal program in southern California. Logar et al. (2014) report findings from a choice experiment, aimed at giving monetary value to benefits deriving from reducing environmental risks of specific micropollutants, including, among others, diclofenac. These benefits are then entered in a cost benefit analysis for the Swiss national plan to reduce micropollutants in treated wastewater effluents, resulting in a positive net present value of this policy.

In another context, Molinos-Senante et al. (2013a, b) quantified the environmental benefits of preventing the discharge of DCF and EE2 into water bodies using the distance function approach. They estimated the shadow prices of those compounds, which can be interpreted as the economic value of environmental benefits to avoid the discharge of contaminants into the environment. In particular, for nonsensitive areas, the shadow prices of the DCF and EE2 were quantified by 42.20 and 73.73 € kg^−1^, while for sensitive areas, they were 53.47 and 93.76 € kg^−1^, respectively. These figures represent the positive externalities of removing both pollutants from wastewater with the highest available standards. For example, the value of 42.20 € kg^−1^ means that for each kilogram of DCF that is removed from wastewater, the environmental benefit is quantified by 42.20 €. It should be noted, that to estimate the overall benefits from wastewater treatment, not only the value of the shadow prices in € per kilogram should be considered, but the volume of each pollutant removed in kilogram per cubic meter as well.

## Concluding remarks

Nearly half of the European countries are facing water stress issues today, both in terms of water scarcity and water quality deterioration, and it is estimated that 20–40 % of Europe’s available water is being wasted (lack of water-saving technologies installed, too much unnecessary irrigation, etc.). In addition, priority and emerging organic pollutants and pathogens are continuously discharged into European rivers and streams, thereby compromising valuable ecosystem services and resulting in potentially adverse effects to aquatic organisms. This is due to the fact that our conventional WWTPs are neither specifically designed nor operated to remove residual concentrations of organic pollutants, causing the potential accumulation of such pollutants into receiving water bodies and limiting at the same time proper reuse of water. Although concentrations of DCF and EE2 in groundwater and surface waters are still generally low and an acute toxicological risk for consumers has not been identified so far, contamination is increasing, like for other emerging compounds.

Cleaning highly treated wastewater through an environmental buffer and stripping residual contaminants into the matrix by sorption and occult microbial processes to augment a drinking water supply is a recent practice, which is referred to as intentional indirect potable reuse, which can occur through recharge of unconfined or confined aquifers. It has been demonstrated by several projects that nonpotable and potable water reuse can represent a viable option to diversify local water resources while at the same time reducing the demand for conventional freshwater supplies. In consequence, the potential economic value of this particular water is decreased.

At this time, there are some novel potent remediation technologies available for conventionally treated wastewater applying, e.g., filtration, adsorption, or ozonation. Typically, they contribute to a substantial removal of pharmaceutical compounds such as DCF and EE2. But only adsorption onto activated carbon and ozonation are techniques which have reached marketable technology readiness levels in Europe at present. Further research needs to be directed into the optimization of such technologies or the transfer of promising technologies and combinations of processes from laboratory to technical scale. WWTP effluents, if properly treated, can be reclaimed and reused for determined restricted uses, contributing in this way to the reduction of water pollutants and the pressure over the worldwide water scarcity. Nevertheless, the use of inadequately treated wastewaters for irrigation will definitely raise public health concerns arising from the presence of microorganisms and contaminants of emerging concern.

This situation strongly calls for the development of optimized remediation techniques to generally limit the release of these substances in the environment. It is also evident that in order to protect resources for future generations, approaches have to be adopted, which sustainably protect ecosystems and biodiversity.

With the increasing need to alleviate the load of emerging contaminants, treatment facilities across Europe need upgrading to fulfill water standards and to keep the end users healthy. The lack of knowledge about the occurrence of many emerging organic pollutants in WWTP effluents as well as about the efficiency of treatment options must be overcome. Interdisciplinary initiatives like the COST Action ESSEM 1202 (Conceiving Wastewater Treatment in 2020—Energetic, environmental and economic challenges; http://www.water2020.eu) are a potent instrument to collect knowledge and feed it into discussion panels. From starting points like those, it will be urgently required to develop overall evaluation schemes for wastewater management, including its energetic, environmental, and economic challenges, to provide national and EU authorities with useful and reliable decision support tools for future investments and implementations. Among these tools, validated sampling and analytical techniques, (eco-)toxicological assessment, and sound economical background data have to be made available. Existing WWTPs need to be upgraded by most advanced modules, enhancing degradation and optimizing overall retention times.

And last but not least, for groundwater, one of our most valuable resources, in addition to the requirements of good status, any significant and sustained upward trend in the concentration of any pollutant should be identified and reversed as early as possible.

## Electronic supplementary material

Below is the link to the electronic supplementary material.

Supplemental Table 1 (DOC 49 kb)
