# Supplementary material for: Status of hormones and painkillers in wastewater effluents across several European states—considerations for the EU watch list concerning estradiols and diclofenac
Source: Environ Sci Pollut Res Int. 2016 Mar 29;23:12835–66. doi: 10.1007/s11356-016-6503-x (PMC4912981; doi:10.1007/s11356-016-6503-x)
Supplement: Supplementary file 1 — (DOC 49 kb) [file 11356_2016_6503_MOESM1_ESM.doc]

Supplemental Data

Table 1. Annual DCF and EE2 consumption in several EU countries. Databases and publications from 2005 to 2013 have been considered.

| Country | DCF  DDD 100 mg | | EE2  DDD 25 µg | | Reference |
| --- | --- | --- | --- | --- | --- |
|  | DDD/1000 inhab/d | mg/inhb/yr | DDD/1000 inhab/d | µg/inhb/yr |  |
| Austria | 21 | 768 | 54.5 | 500 | Kreuzinger et al. 2004 |
| Estonia | 13.1 | 480 | 24.2 | 220 | *Estonian State Agency of Medicines 2013* |
| Finland | 4.2 | 154 | 2.2 | 20 | Alder et al. 2006 |
| France | 6.9 | 255 | n.d. | n.d. | Alder et al. 2006 |
| Germany | 28.2 | 1033 | 66 | 600 | SRU, 2007 |
| Italy | 4.3 | 160 | 15.9 | 150 | <http://www.epicentro.iss.it/farmaci/OsMed.asp> |
| Lithuania | 20.5 | 750 | 12.2 | 111 | *Estonian State Agency of Medicines 2013* |
| Latvia | 23.9 | 870 | 11.4 | 104 |  |
| Netherlands | 12 | 440 | n.a. | n.a. | Oosterhus et al. 2012 |
| Norway | 11.5 | 420 | 38.7 | 353 | <http://www.norpd.no/Prevalens.aspx> |
| Poland | 14.7 | 540 | 22.9 | 210 | Alder et al. 2006 |
| Portugal | 10.1 | 370 | 6.0 | 55 | INFARMED 2012 |
| Serbia | 34.7 | 1260 | 6.67 | 60 | Radonjic and Sipetic 2012 |
| Spain | 14.4 | 541 | 22.9 | 210 | Carballa et al. 2008 |
| Sweden | 10.6 | 390 | 29.2 | 266 | <http://www.socialstyrelsen.se/statistik/statistikdatabas/lakemedel> |
| Turkey | 26.9 | 985 | n.a. | n.a. |  |
